# The lncRNA *MALAT1* is upregulated in urine of type 1 diabetes mellitus patients with diabetic kidney disease

**DOI:** 10.1590/1678-4685-GMB-2022-0291

**Published:** 2023-06-02

**Authors:** Cristine Dieter, Natália Emerim Lemos, Eliandra Girardi, Denise Taurino Ramos, Nathalia Rodrigues de Faria Corrêa, Luís Henrique Canani, Andrea Carla Bauer, Taís Silveira Assmann, Daisy Crispim

**Affiliations:** 1 Hospital de Clínicas de Porto Alegre, Serviço de Endocrinologia e Metabologia, Porto Alegre, RS, Brazil.; 2 Universidade Federal do Rio Grande do Sul, Faculdade de Medicina, Departamento de Medicina Interna, Programa de Pós-Graduação em Ciências Médicas: Endocrinologia, Porto Alegre, RS, Brazil.; 3 Universidade de São Paulo, Instituto de Química, Departamento de Bioquímica, São Paulo, SP, Brazil.; 4 Hospital de Clínicas de Porto Alegre, Serviço de Nefrologia, Porto Alegre, RS, Brazil.

**Keywords:** lncRNAs, MALAT1, TUG1, diabetic kidney disease, biomarker.

## Abstract

Long non-coding RNAs (lncRNAs) are RNAs with >200 nucleotides that are unable to encode proteins and are involved in gene expression regulation. LncRNAs have a key role in many physiological and pathological processes and, consequently, they have been associated with several human diseases, including diabetes chronic complications, such as diabetes kidney disease (DKD). In this context, some studies have identified the dysregulation of the lncRNAs *MALAT1* and *TUG1* in patients with DKD; nevertheless, available data are still contradictory. Thus, the objective of this study was to compare *MALAT1* and *TUG1* expressions in urine of patients with type 1 diabetes mellitus (T1DM) categorized according to DKD presence. This study comprised 18 T1DM patients with DKD (cases) and 9 long-duration T1DM patients without DKD (controls). *MALAT1* and *TUG1* were analyzed using qPCR. Bioinformatics analyses were done to identify both lncRNA target genes and the signaling pathways under their regulation. The lncRNA *MALAT1* was upregulated in urine of T1DM patients with DKD *vs*. T1DM controls (P = 0.007). The expression of lncRNA *TUG1* did not differ between groups (P = 0.815). Bioinformatics analysis showed these two lncRNAs take part in metabolism-related pathways. The present study shows that the lncRNA *MALAT1* is upregulated in T1DM patients presenting DKD.

## Introduction

Diabetic kidney disease (DKD) is a common severe microvascular complication of diabetes mellitus (DM), usually leading to increased morbidity and mortality rates in DM patients (Samsu, 2021). DKD is characterized by clinical manifestations, such as albuminuria and a progressive decline in the glomerular filtration rate (GFR), which may progress to end-stage renal disease (ESRD) (Ritz et al., 2011). Pathological characteristics of this complication comprise glomerular mesangial expansion and hypertrophy, tubular interstitial fibrosis, glomerular sclerosis, apoptosis of podocytes, and deposition of extracellular matrix (ECM) proteins (Reidy et al., 2014; Akhtar et al., 2020).

The main risk factors for DKD development are the chronic hyperglycemia and high blood pressure (Samsu, 2021). Recent studies have also highlighted the key involvement of epigenetics factors, such as long non-coding RNAs (lncRNAs), in the pathogenesis of DKD (Zhao et al., 2022). LncRNAs are non-coding RNAs (ncRNAs) with at least 200 nucleotides in length and unable to codify proteins. They have key roles in different physiological functions and pathological mechanisms, regulating gene expression at the transcriptional, posttranscriptional, and epigenetic levels (Kaikkonen and Adelman, 2018), Moreover, lncRNAs are known to be involved in the differentiation, proliferation, and death of many cell types (Kaikkonen and Adelman, 2018).

Different lncRNAs seem to be altered in DKD patients [reviewed in (Zhao et al., 2022)]. The lncRNA *metastasis-associated ling adenocarcinoma transcript 1* (*MALAT1*) was upregulated in peripheral blood mononuclear cells (PBMCs) from T2DM patients with DKD compared to the control group (Zhou et al., 2020). Accordingly, expression of *Malat1* was augmented in kidneys of C57BL/6 mice with DKD induced by streptozotocin (STZ) treatment (Hu et al., 2017). In the DKD context, some studies have also reported that alterations in *MALAT1* expression were associated with cell viability, apoptosis, inflammatory response, and cell injury pathways (Song et al., 2022; Yang et al., 2022; Shoeib et al., 2023). Moreover, downregulation of the lncRNA *taurine-upregulated gene 1* (*TUG1*) possibly contributes to the progress of DKD by activating endoplasmic reticulum stress and podocyte apoptosis (Shen et al., 2019). Interestingly, *TUG1* upregulation reduced the production of ECM proteins and inhibited cell proliferation in STZ-induced DM rats as well as in high glucose (HG)-treated mesangial cells (MCs)*via*inhibition of PI3K/AKT pathway. Thus, *TUG1* upregulation could hinder the evolution of DKD to its severe forms (Zang et al., 2019).

Taking these studies into consideration, *MALAT1* and *TUG1* seem to be involved in DKD pathogenesis, although their specific roles are unknown. Thus, through a case-control design, we analyzed *MALAT1* and *TUG1* expressions in urine from patients with type 1 DM (T1DM) categorized according to DKD presence. We also performed bioinformatics analyses to explore the target genes and signaling pathways possibly regulated by these two lncRNAs.

## Material and Methods

### Samples and clinical and laboratory evaluations

The STROBE guidelines were used to design and implement this case-control study (von Elm et al., 2014). Twenty-seven T1DM patients were categorized into nine patients without DKD (control group) and 18 cases with DKD. Patients were from Instituto da Criança com Diabetes at Grupo Hospitalar Conceição (Rio Grande do Sul, Brazil), and were recruited between November 2019 and May 2022. American Diabetes Association guidelines were followed for T1DM diagnosis (American Diabetes Association, 2018). 

The patients were classified using the estimated glomerular filtration rate (eGFR) according to Kidney Disease Improving Global Outcomes (KDIGO) guidelines (Andrassy, 2013). The eGFR values were calculated with the CKD-EPI equation (Levey *et al.*, 2009). Patients with eGFR ≥90 ml/min/1.73 m² and ≥10 years of T1DM were classified as controls, while patients with eGFR <90 ml/min/1.73 m² were classified as DKD cases. 

Presence of febrile episodes in the last 3 months, inflammatory or rheumatic diseases, HIV-positivity, hepatitis, liver or cardiac failure, kidney transplantation, hereditary dyslipidemia, errors of metabolism excepting DM, and glucocorticoid treatment were the exclusion criteria. Since the period of the day might influence lncRNA expression, samples were collected in the morning for all patients.

A questionnaire was applied to retrieve data on age, age at diagnosis, T1DM duration, ethnicity, and drug treatment. Ethnicity classification was based on self-classification. All subjects were submitted to both physical and laboratory tests, as previously described (Assmann et al., 2014). Serum creatinine levels were evaluated using the Jaffé reaction (Zelmanovitz et al., 1997). Written informed consents were obtained from all patients before inclusion in the study, and the study was approved by the Ethic Committees in Research from Hospital de Clínicas de Porto Alegre and Grupo Hospitalar Conceição/Instituto da Criança com Diabetes.

### RNA extraction

Voided midstream urine samples (20 mL) were collected from patients, centrifuged at 3200 × *g* for 5 min at 4 °C, and then aliquoted and stored at -80 °C until analysis of lncRNA expressions. Total RNA was extracted from 200 µL urine samples using the miRNeasy Serum/Plasma Kit (Qiagen, Hilden, Germany). RNA purity and concentration parameters were analyzed in the NanoDrop ND-1000 Spectrophotometer (Thermo Fisher Scientific, Waltham, MA, USA). Those RNAs that did not achieve suitable purity ratios (A260/A280 = 1.9-2.1) were excluded from gene expression analysis (Bustin et al., 2009). 

### Quantification of lncRNA expressions by RT-qPCR

Reverse transcription real-time quantitative PCR (RT-qPCR) reactions were done in two separate steps: 1) total RNAs were reverse-transcribed into cDNA; and 2) cDNA samples were amplified by qPCR. Reverse transcription was performed using the SuperScript VILO Master Mix IV (Thermo Fisher Scientific). cDNA samples were then amplified by qPCR, which was run in a ViiAÔ 7 Fast Real-Time PCR System (Thermo Fisher Scientific). Each PCR reaction contained 0.5 µL TaqMan Gene Expression Assay (20x) (Thermo Fisher Scientific) for *MALAT1* (assay ID: Hs00273907_s1) and *TUG1* (assay ID: Hs05579214_s1) or the reference gene (*GAPDH* assay ID: Hs02786624_g1), 5 µL TaqMan Fast Advanced Master Mix (Thermo Fisher Scientific), and 1 µL of cDNA (150 ng/µl for *TUG1* and 70 ng/µl for *MALAT1*) plus sterile water to complete 10 µL. Samples were analyzed in triplicates and three negative controls were included in each qPCR plate. Cycling steps were as follows: an initial cycle of 50 °C (2 min), a second cycle of 95 °C (10 min), plus 45 cycles of 95 °C (1 s) and 60 °C (20 s). Quantifications of the two lncRNAs were performed using the 2^-ΔΔCq^ method and the *GAPDH* gene as the reference and are shown as n-folds in relation to the calibrator sample (Bustin et al. 2009). The reference gene was selected after we tested the expression of *GAPDH*, *ACTB*, *PPIA* (CYPA), and *TBP* in our samples. *GAPDH* showed the lowest variation between samples and groups and, thus, was selected as the reference gene. The calibrator sample was constituted by a mixture of all cDNAs from the samples included in the study.

### Bioinformatics analysis

The starBase database was used to identify target genes of the two analyzed lncRNAs (Li et al., 2014). Statistical significances were calculated after correcting for multiple comparisons using the Benjamini-Hochberg test and are shown as q-values (Benjamini and Hochberg 1995). A network analysis was performed using the PathDIP (accessed 26^th^ July 2022) to assess the biological significances of lncRNA target genes (Rahmati et al., 2017). Subcellular locations of lncRNAs were investigated using the RNALocate (Zhang T et al., 2017), iLoc-lncRNA (Su *et al.*, 2018), and lncLocator (Cao et al., 2018) online tools. LncRNA and mRNA names were unified following the LNCipedia v5.2 and HUGO gene nomenclature committee (HGNC), respectively. 

### Statistical analysis

Variables with normal distributions are shown as mean ± standard deviation (SD), while variables with skewed distributions were log-transformed and then showed as median (25-75^th^ percentiles). Categorical variables are shown as %. Variables related to clinical and laboratory data and lncRNA expressions were compared between case and control groups using One-way ANOVA, Student’s*t* or χ^2^ tests. Spearman’s tests were used to evaluate correlations between quantitative variables. Statistical analyses were carried out using the SPSS statistical package (v.18.0) for Windows (SPSS Inc, Chicago, IL). Statistical significance was considered when P values were lower than 0.05.

Using the OpenEpi web tool (https://www.openepi.com), we calculated that at least 9 patients in each group were required to have adequate statistical power (β = 80% and α = 0.05) to detect 2 fold change (± 1.5 SD) differences in lncRNA expressions between groups.

## Results

### Characteristics of the sample

Table 1 shows main clinical and laboratory characteristics of the patients with T1DM (cases *vs*. controls). Males comprised 44.4% of the control group and 27.8% of the cases. Mean age (± SD) was 33.4 (± 5.5) in cases and 31.3 (± 6.4) in the control group. Frequency of hypertension was 50.0% in DKD cases and 22.2% in the T1DM control group. As expected, creatinine levels were higher while eGFR values were lower in DKD patients *vs*. T1DM controls. 

**Table 1 -:** Clinical and laboratory characteristics of T1DM controls and DKD cases.

Characteristic	T1DM controls (n = 9)	DKD cases (n = 18)
Age (years)	31.3 ± 6.4	33.4 ± 5.5
Gender (% male)	44.4	27.8
Ethnicity (% black)	11.1	22.2
BMI (kg/m²)	28.9 ± 4.7	28.2 ± 4.4
HbA1c (%)	7.7 (7.5 - 8.3)	8.2 (7.5 - 10.1)
Hypertension (%)	22.2	50.0
Duration of T1DM (years)	24.4 ± 9.5	22.4 ± 6.5
Creatinine (mg/dl)	0.7 (0.6 - 0.8)	1.2 (1.0 - 1.7)
eGFR (mL/min per 1.73m2)	117.0 ± 10.5	62.3 ± 24.4
Diabetic retinopathy (%)	33.3	38.9

Variables are shown as mean ± SD, median (25^th^-75^th^ percentiles) or %. BMI: body mass index; DKD: diabetic kidney disease; eGFR: estimated glomerular filtration rate; HbA1c: glycated hemoglobin; T1DM: type 1 diabetes mellitus.

### *MALAT1* and *TUG1* expressions in urine of T1DM patients with and without DKD

**Figure 1 -:**
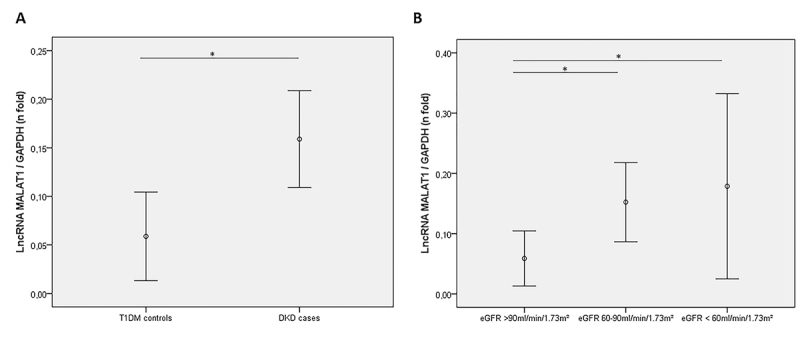
*MALAT1* expression in urine of T1DM patients without DKD (controls) and T1DM patients with DKD (cases). (**A**) *MALAT1* expression between control and case groups. (**B**) *MALAT1* expression between controls and cases with eGFR values of 60 to 90 ml/min/1.73 m² and DKD cases with eGFR <60 ml/min/1.73 m². Relative expression was quantified with RT-qPCR experiments. Data are shown as fold changes relative to the calibrator (ΔΔCq method) and are presented as median (25-75th percentiles). P-values were obtained from ANOVA or Student’s*t*tests, as applicable.^*^P < 0.050.

*MALAT1* expression was higher in DKD patients compared to T1DM control patients [0.140 (0.120 - 0.198) *vs.* 0.065 (0.250 - 0.089), P = 0.007, Figure 1A]. Moreover, when we analyzed its expression according to eGFR values, *MALAT1* was upregulated in both those patients with eGFR between 60 to 90 ml/min/1.73 m² and patients with eGFR <60 ml/min/1.73 m² compared to T1DM control patients [eGFR 60-90 ml/min/1.73 m² group: 0.136 (0.099 - 0.185); eGFR <60 ml/min/1.73 m² group: 0.147 (0.139 - 0.250); control group: 0.065 (0.025 - 0.089); P = 0.013, Figure 1B]. No difference was found in lncRNA *TUG1* expression between cases and controls (P = 0.815) or between controls and patients with eGFR between 60 to 90 ml/min/1.73 m² and patients with eGFR <60 ml/min/1.73 (P = 0.973) (Figure S1).

Next, we analyzed correlations between *MALAT1* and *TUG1* expressions in urine and eGFR and creatinine values in all T1DM patients. *MALAT1* expression showed a negative correlation with eGFR values (r = -0.555, P = 0.021). Moreover, *MALAT1* expression seems to be positively correlated with creatinine levels; but this analysis did not achieve formal significance (r = 0.464, P = 0.060). *TUG1* expression was not correlated with DKD-related measurements (P > 0.050). 

### Target genes and enrichment pathway analysis for *MALAT1* and *TUG1*

Bioinformatics analyses were done to identify possible target genes of *MALAT1* and *TUG1*. Together these two lncRNAs regulate the expression of 1,815 genes (Table S1). *MALAT1*has 1,598 target genes while *TUG1* has 295 target genes. Among the 1,815 targets, 1,231 encode proteins, 319 are pseudogenes, 102 are small nuclear RNA (snRNA) genes, and 163 are other ncRNAs, such as microRNAs, mitochondrial RNA, rRNAs, and tRNAs (Table S1). 

In order to explore in better details the functional significances of these two lncRNAs, we next carried out functional enrichment analysis of their targets using the KEGG repository. This analysis identified 79 pathways that were enriched for the lncRNA targets. Some of the 79 pathways are already acknowledged as having a key role in DM and DKD pathogenesis, including the glycolysis/gluconeogenesis, PI3K-Akt, AMPK, and type 1 DM pathways (Table S2). 

### LncRNA localization

We also searched the subcellular localization of the two lncRNAs investigated in T1DM patients. The RNAlocate database comprises manually curated subcellular localization data of RNAs derived from experimental studies. The iLoc-lncRNA and lncLocator tools predicts RNA subcellular locations based on RNA sequence. Based on the iLoc-lncRNA score, the lncRNA *MALAT1* is located in the nucleolus, nucleus, and nucleoplasm (Table 2). LncLocator also indicated the presence of this lncRNA in cytoplasm and nucleus. Regarding *TUG1*, according to iLoc-lncRNA and lncLocator, its subcellular location is cytoplasm and cytosol (Table 2). Nevertheless, in relation to RNALocate database information, we observed that ncRNA localization may vary across different tissues, cells or conditions in which they are expressed (Table 2).

**Table 2-:** Subcellular location of the lncRNAs *MALAT1* and *TUG1* according to three different databases/tools.

A, iLoc lncRNA tool		
lncRNA	Subcellular location	Probability score
*MALAT1*	Nucleolus, nucleus, nucleoplasm	0.517962
*TUG1*	Cytoplasm, cytosol	0.857667
B, lncLocator tool		
lncRNA	Subcellular location	Probability score
*MALAT1*	Cytoplasm	0.620778
	Nucleus	0.304726
	Ribosome	0.009907
	Cytosol	0.050084
	Exosome	0.014504
*TUG1*	Cytoplasm	0.835823
	Nucleus	0.131329
	Ribosome	0.008885
	Cytosol	0.019812
	Exosome	0.004149
C, RNALocate		
lncRNA	Subcellular location	Tissue
*MALAT1*	Chromatin	Breast cancer cell line, HeLa-S3 cells, K562 cells
	Cytoplasm	Lung cancer cell lines, HCC cell line, HeLa-S3 cells, K562 cells
	Exosome	Thyroid papillary carcinoma cell line, HeLa cells, serum
	Insoluble cytoplasm	K562 cells
	Membrane	HCC cell line, K562 cells
	Mitochondrion	HCC cell line
	Nuclear speckle	Breast cancer cell line, retinal microvascular endothelial cells, U2OS cells, HEK-239T cells, mammary epitheliem cell line, HeLa-TO cells
	Nucleolus	K562 cells
	Nucleoplasm	WI-38 cells, K562 cells
	Nucleus	Human osteosarcoma cell line, HeLa cells, WI-38 cells, human brain microvascular endothelial cells, breast cancer cell lines, lung cancer cell line, vascular smooth muscle cells, fibroblasts, lymphoblasts, motor neurons, CLTon cells, HCC cell line, K562 cells
	Speckle periphery	HeLa cells, WI-38 cells
C, RNALocate		
lncRNA	Subcellular location	Tissue
*TUG1*	Cytoplasm	CC cell line, hES/iPS cells
	Cytosol	HCC cell line
	Exosome	Serum
	Insoluble cytoplasm	K562 cells
	Membrane	HCC cell line
	Nucleolus	K562 cells
	Nucleoplasm	K562 cells
	Nucleus	HCC cell line, hFF cells, hLF cells, HeLa cells, hES/iPS cells

## Discussion

Proteinuria and progression of DKD may be influenced by dysregulated lncRNA expressions. Therefore, to better understand the involvement of lncRNAs *MALAT1* and *TUG1* in DKD, we analyzed their expressions in T1DM patients categorized according to DKD presence. *MALAT1* was upregulated in urine from patients with DKD compared to those patients without DKD. Moreover, *MALAT1* expression showed a negative correlation with eGFR levels. No difference was observed in *TUG1* levels between case and control groups. 

*MALAT1*, also referred as *NEAT2*, is located in the human chromosome 11q13 and acts as an oncogene in many cancers (Zhang X et al., 2017; Arun et al., 2020). *MALAT1* seems to trigger inflammation and oxidative stress, which are key processes involved in the development of DKD, by upregulating a number of inflammatory molecules (Puthanveetil et al., 2015; Li et al., 2019). Moreover, *MALAT1* is involved in podocyte damage and renal fibrosis (Hu et al., 2017; Arun *et al.*, 2020; Huang et al. 2021). 

In accordance with our results, other studies demonstrated an increased *MALAT1* expression in DKD patients. Zhou et al. (2020) showed an increase of this lncRNA in PBMCs of T2DM patients with DKD compared to T2DM controls and healthy individuals as well as its positive correlation with creatinine levels in T2DM patients (Zhou *et al.*, 2020), which was also observed in our study. Higher expression of lncRNA *MALAT1* was also observed in DM patients with ESRD *vs*. DM controls (Fawzy et al., 2020). In addition, urinary and serum levels of *MALAT1* were reported as being increased in DKD patients compared to DM controls and healthy subjects (Petrica *et al.*, 2021). Additionally, this lncRNA showed a negative correlation with eGFR in both plasma and urine samples of T2DM patients (Petrica *et al.*, 2021), which is in accordance with our data. 

Experimental studies also reported *Malat1* upregulation in the renal context. *Malat1* upregulation was reported in renal tubular epithelium of STZ-induced diabetic rats compared to control rats and in human renal epithelial cell lines treated with HG (Huang et al., 2021). Moreover, the authors suggested that *Malat1* upregulation is able to increase renal fibrosis in diabetic rats and damage HG-incubated HK-2 cells by acting through the miR-2355-3p/IL6ST pathway (Huang *et al.*, 2021). Zhang et al. (2021) demonstrated the upregulation of *Malat1* in renal tissues of a murine model of DKD (db/db) and podocytes MPC5 cells treated with HG compared to controls. Silencing of *Malat1* suppressed the damage of podocytes as well as the inflammation and oxidative stress in kidneys of DKD mice (Zhang *et al.*, 2021). *Malat1* upregulation was also observed in the renal cortex from a model of STZ-induced T1DM mice (C57BL/6) as well as mouse podocytes stimulated with HG compared to controls (Hu *et al.*, 2017). Hence, this lncRNA may have a role in the progression of DKD and is a great candidate to be used as a DKD biomarker. 

LncRNA *TUG1* has been involved in various physiological functions, including cell proliferation, migration and death, and regulation of cell cycle (reviewed in Guo et al., 2020). In the context of renal damage, *TUG1* seems to be involved in podocyte apoptosis and effacement (Shen et al., 2019; Lei *et al.*, 2022), which are involved in glomerular dysfunction and proteinuria. This lncRNA was reported as being downregulated in podocytes of T2DM db/db mice compared to control animals and also in the glomeruli of DKD patients (Long et al., 2016). Long *et al.* (2016) demonstrated *TUG1* downregulation in podocytes of diabetic mice and its interaction with Pgc-1α, which has a key role in the transcriptional regulation of mitochondrial biogenesis. Moreover, overexpression of *Tug1*in podocytes was able to upregulate *Pgc-1*α expression*,* leading to improved mitochondrial bioenergetics (Long *et al.*, 2016). Hence,*Tug1*downregulation seems to decrease *PGC-1*α expression and its downstream genes, consequently influencing mitochondrial biogenesis and then leading to apoptosis of podocyte cells and glomerular dysfunction (Long *et al.*, 2016; Tanwar et al., 2021).

In humans, *TUG1* expression was downregulated in urine and serum samples of DKD patients compared to T2DM patients without DKD (Petrica *et al.*, 2021). Moreover, *TUG1* serum and urinary expressions correlated positively with eGFR (Petrica *et al.*, 2021). To our knowledge, no other study has investigated *TUG1* expression in human samples from DM patients with or without DKD. Thus, considering that we did not observe any significant difference in the expression of this lncRNA between groups, more studies are required to confirm the dysregulation of *TUG1* found by Petrica *et al.* (2021).

Moreover, our bioinformatics analysis showed that *MALAT1* and TUG1 target genes are involved in DM and DKD related-pathways, such as glycolysis/gluconeogenesis, PI3K-Akt, AMPK, type 1 DM, Wnt, and TGF-beta. In addition, it is known that the subcellular localization of lncRNAs may complement information about the structural characteristics and different functions of these ncRNAs (Biswas et al., 2022), which might affect susceptibility to DKD. Despite this, the exact localization of lncRNAs remains controversial and there is a lack of information regarding the localization of these two lncRNAs in the context of DM and its complications. Hence, our bioinformatics analyses suggest possible localizations of *MALAT1* and *TUG1*.

Although our results are important to complement the role of *MALAT1* and *TUG1* in DKD pathogenesis, we have to draw attention to a few limitations. We cannot dismiss the occurrence of type II errors during comparisons of lncRNA expressions between study groups, but the chance of this type of error has been reduced considering that our sample size has enough statistical power to detect two fold change differences in lncRNA expressions between the analyzed groups. Moreover, a number of variables can influence lncRNA expressions. To reduce the effect of these variables on our data, we have opted to apply a broad list of exclusion criteria to our patients. Even though these limitations, our results are important to be described considering this is the first report of *MALAT1* and *TUG1* expressions in urinary samples from Brazilian T1DM patients divided according to DKD occurrence.

In conclusion, our study shows the upregulation of *MALAT1* in urine of T1DM patients with DKD in comparison to T1DM patients without DKD. Additionally, we suggest that *MALAT1* expression in urine could be used as a candidate biomarker for DKD since it is associated with renal damage and correlated with renal markers, such as eGFR and creatinine. 

## Supplementary material

The following online material is available for this article:

Figure S1 -
*TUG1* expression in urine of T1DM patients without DKD (controls) and T1DM patients with DKD (cases).

Table S1 - Target genes of the lncRNAs *MALAT1* and *TUG1* investigated in T1DM patients.

Table S2 - Significant KEGG pathways regulated by the target genes of the lncRNAs *MALAT1* and *TUG1*.
