# Supplementary material for: The lncRNA MALAT1 is upregulated in urine of type 1 diabetes mellitus patients with diabetic kidney disease
Source: Genet Mol Biol. 2023 Jun 2;46(2):e20220291. doi: 10.1590/1678-4685-GMB-2022-0291 (PMC10240573; doi:10.1590/1678-4685-GMB-2022-0291)
Supplement: Figure S1 - [file 1415-4757-GMB-46-2-e20220291-s1.pdf]

Genetics and Molecular Biology
DOI: https://doi.org/10.1590/1678-4685-GMB-2022-0291

Supplementary Material to "The lncRNA *MALAT1* is upregulated in urine of type 1 diabetes mellitus patients with diabetic kidney disease"

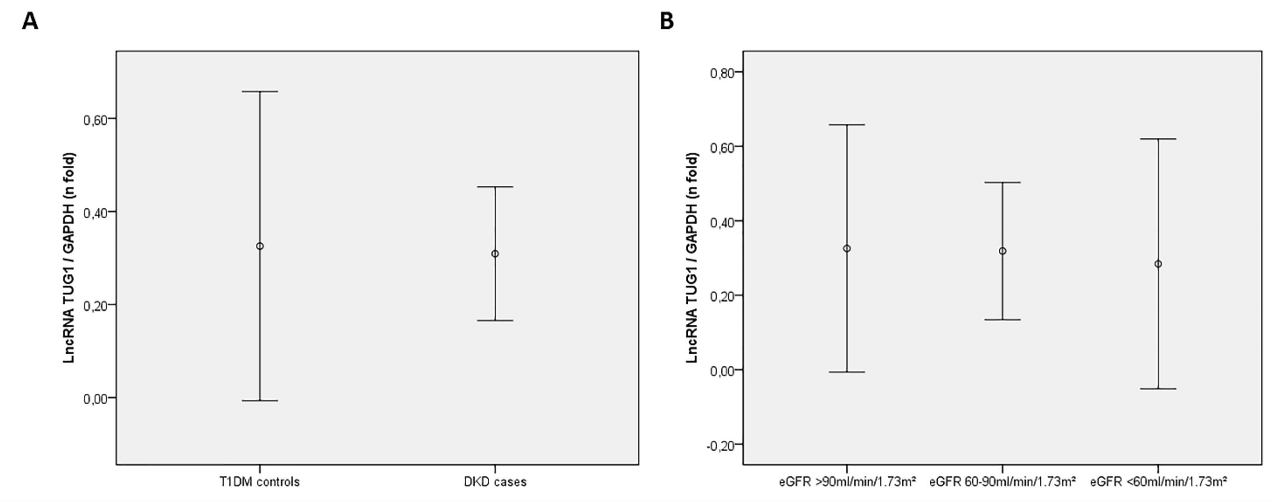

Figure S1 - *TUG1* expression in urine of T1DM patients without DKD (controls) and T1DM patients with DKD (cases). (**A**) *TUG1* expression between control and case groups. (**B**) *TUG1* expression between controls and cases with eGFR values of 60 to 90 ml/min/1.73 m^2 and DKD cases with eGFR <60 ml/min/1.73 m^2. Relative expressions were quantified with RT-qPCR experiments. Data are shown as fold changes relative to the calibrator (ΔΔCq method), and are presented as median (25–75th percentiles). P values were obtained from ANOVA or Student's *t* tests, as applicable. *P < 0.050.
