# Supplementary material for: The lncRNA MALAT1 is upregulated in urine of type 1 diabetes mellitus patients with diabetic kidney disease
Source: Genet Mol Biol. 2023 Jun 2;46(2):e20220291. doi: 10.1590/1678-4685-GMB-2022-0291 (PMC10240573; doi:10.1590/1678-4685-GMB-2022-0291)
Supplement: Table S1 - [file 1415-4757-GMB-46-2-e20220291-s2.pdf]

Genetics and Molecular Biology
DOI: https://doi.org/10.1590/1678-4685-GMB-2022-0291

Supplementary Material to "The lncRNA *MALAT1* is upregulated in urine of type 1 diabetes mellitus patients with diabetic kidney disease"

Table S1 - Target genes of the lncRNAs *MALAT1* and *TUG1* investigated in T1DM patients.

lncRNA	*Target*	Gene type
MALAT1	*PLXND1*	protein_coding
MALAT1	*AK2*	protein_coding
MALAT1	*GDE1*	protein_coding
MALAT1	*TMEM132A*	protein_coding
MALAT1	*MGST1*	protein_coding
MALAT1	*ZNF207*	protein_coding
MALAT1	*AKAP8L*	protein_coding
MALAT1	*PTBP1*	protein_coding
MALAT1	*SYT7*	protein_coding
MALAT1	*ANLN*	protein_coding
MALAT1	*WIZ*	protein_coding
MALAT1	*ERCC1*	protein_coding
MALAT1	*TACC3*	protein_coding
MALAT1	*CAPN1*	protein_coding
MALAT1	*MDH1*	protein_coding
MALAT1	*STRAP*	protein_coding
MALAT1	*NCAPH2*	protein_coding
MALAT1	*ADSS*	protein_coding
MALAT1	*RIPOR1*	protein_coding
MALAT1	*PSMA4*	protein_coding
MALAT1	*CTNNA1*	protein_coding
MALAT1	*GUCA1A*	protein_coding
MALAT1	*LIMA1*	protein_coding
MALAT1	*TTC17*	protein_coding
MALAT1	*AKR7A2*	protein_coding
MALAT1	*AP5M1*	protein_coding
MALAT1	*PPP1R12A*	protein_coding
MALAT1	*CCAR1*	protein_coding
MALAT1	*APPBP2*	protein_coding
MALAT1	*HIPK2*	protein_coding
MALAT1	*BORCS8-MEF2B*	protein_coding
MALAT1	*ADAT1*	protein_coding
MALAT1	*TMEM206*	protein_coding
MALAT1	*ISOC1*	protein_coding
MALAT1	*SP100*	protein_coding
MALAT1	*PKM*	protein_coding
MALAT1	*RHOA*	protein_coding

lncRNA	*Target*	Gene type
MALAT1	*DGCR2*	protein_coding
MALAT1	*CSNK2A2*	protein_coding
MALAT1	*SNX13*	protein_coding
MALAT1	*TRIP13*	protein_coding
MALAT1	*PDCD2*	protein_coding
MALAT1	*HMMR*	protein_coding
MALAT1	*CLNS1A*	protein_coding
MALAT1	*ZZEF1*	protein_coding
MALAT1	*ENO1*	protein_coding
MALAT1	*TTC38*	protein_coding
MALAT1	*MARK3*	protein_coding
MALAT1	*ACTB*	protein_coding
MALAT1	*BCAP29*	protein_coding
MALAT1	*FBLN1*	protein_coding
MALAT1	*GNB1*	protein_coding
MALAT1	*SENP1*	protein_coding
MALAT1	*DNM2*	protein_coding
MALAT1	*EPB41L2*	protein_coding
MALAT1	*SMARCA2*	protein_coding
MALAT1	*NFE2L1*	protein_coding
MALAT1	*NOA1*	protein_coding
MALAT1	*MAP3K4*	protein_coding
MALAT1	*IGSF9*	protein_coding
MALAT1	*PGS1*	protein_coding
MALAT1	*GNAS*	protein_coding
MALAT1	*CNOT3*	protein_coding
MALAT1	*KHSRP*	protein_coding
MALAT1	*ANKRD10*	protein_coding
MALAT1	*C20orf194*	protein_coding
MALAT1	*FUS*	protein_coding
MALAT1	*GANAB*	protein_coding
MALAT1	*BIRC5*	protein_coding
MALAT1	*MLF2*	protein_coding
MALAT1	*NECAP1*	protein_coding
MALAT1	*PCBP4*	protein_coding
MALAT1	*TFAP4*	protein_coding
MALAT1	*HNRNPC*	protein_coding
MALAT1	*EZR*	protein_coding
MALAT1	*UPRT*	protein_coding
MALAT1	*ARCN1*	protein_coding
MALAT1	*WAC*	protein_coding
MALAT1	*FKBP5*	protein_coding
MALAT1	*DSP*	protein_coding
MALAT1	*CDC7*	protein_coding
MALAT1	*SCD*	protein_coding
MALAT1	*ERMP1*	protein_coding
MALAT1	*CRKL*	protein_coding

lncRNA	*Target*	Gene type
MALAT1	*DDX17*	protein_coding
MALAT1	*GTPBP1*	protein_coding
MALAT1	*RPL3*	protein_coding
MALAT1	*COCH*	protein_coding
MALAT1	*PSMC6*	protein_coding
MALAT1	*VTI1B*	protein_coding
MALAT1	*TCL1A*	protein_coding
MALAT1	*ADNP*	protein_coding
MALAT1	*HM13*	protein_coding
MALAT1	*ACOT8*	protein_coding
MALAT1	*PGK1*	protein_coding
MALAT1	*ARMCX3*	protein_coding
MALAT1	*CCL22*	protein_coding
MALAT1	*COG4*	protein_coding
MALAT1	*NAGPA*	protein_coding
MALAT1	*COTL1*	protein_coding
MALAT1	*ZNF500*	protein_coding
MALAT1	*NUBP1*	protein_coding
MALAT1	*GSPT1*	protein_coding
MALAT1	*DNAJA3*	protein_coding
MALAT1	*SFRP1*	protein_coding
MALAT1	*GPI*	protein_coding
MALAT1	*HNRNPUL1*	protein_coding
MALAT1	*CD33*	protein_coding
MALAT1	*GRWD1*	protein_coding
MALAT1	*ZNF175*	protein_coding
MALAT1	*PPP2R1A*	protein_coding
MALAT1	*RPL18A*	protein_coding
MALAT1	*ISYNA1*	protein_coding
MALAT1	*ZKSCAN1*	protein_coding
MALAT1	*COA1*	protein_coding
MALAT1	*UBE2R2*	protein_coding
MALAT1	*PLEKHA1*	protein_coding
MALAT1	*UNC5B*	protein_coding
MALAT1	*ACTA2*	protein_coding
MALAT1	*ARHGAP21*	protein_coding
MALAT1	*ZMIZ1*	protein_coding
MALAT1	*CASC3*	protein_coding
MALAT1	*CBX1*	protein_coding
MALAT1	*PFN1*	protein_coding
MALAT1	*RNF167*	protein_coding
MALAT1	*DDX5*	protein_coding
MALAT1	*DHX15*	protein_coding
MALAT1	*MTCH2*	protein_coding
MALAT1	*HSPA8*	protein_coding
MALAT1	*LPXN*	protein_coding
MALAT1	*PRPF19*	protein_coding

lncRNA	*Target*	Gene type
MALAT1	*GALNT18*	protein_coding
MALAT1	*UBE4A*	protein_coding
MALAT1	*MDK*	protein_coding
MALAT1	*RPS13*	protein_coding
MALAT1	*CORO1C*	protein_coding
MALAT1	*OAS2*	protein_coding
MALAT1	*SLC38A1*	protein_coding
MALAT1	*CPSF6*	protein_coding
MALAT1	*CDCA3*	protein_coding
MALAT1	*TPI1*	protein_coding
MALAT1	*COX6A1*	protein_coding
MALAT1	*SRSF9*	protein_coding
MALAT1	*MAPK14*	protein_coding
MALAT1	*FIG4*	protein_coding
MALAT1	*MRPL2*	protein_coding
MALAT1	*VEGFA*	protein_coding
MALAT1	*SLC29A1*	protein_coding
MALAT1	*PAPD7*	protein_coding
MALAT1	*HMGCR*	protein_coding
MALAT1	*CCNG1*	protein_coding
MALAT1	*SLC12A7*	protein_coding
MALAT1	*PPP2CA*	protein_coding
MALAT1	*DBN1*	protein_coding
MALAT1	*HEMK1*	protein_coding
MALAT1	*NEK4*	protein_coding
MALAT1	*DGUOK*	protein_coding
MALAT1	*MOB1A*	protein_coding
MALAT1	*TTL*	protein_coding
MALAT1	*ZNF142*	protein_coding
MALAT1	*CNPPD1*	protein_coding
MALAT1	*NFE2L2*	protein_coding
MALAT1	*RALGPS2*	protein_coding
MALAT1	*STXBP3*	protein_coding
MALAT1	*ASH1L*	protein_coding
MALAT1	*ARHGEF2*	protein_coding
MALAT1	*WLS*	protein_coding
MALAT1	*BMP8B*	protein_coding
MALAT1	*SDHB*	protein_coding
MALAT1	*KDM5B*	protein_coding
MALAT1	*AKR1A1*	protein_coding
MALAT1	*IRF6*	protein_coding
MALAT1	*STMN1*	protein_coding
MALAT1	*PPP1R8*	protein_coding
MALAT1	*CTSD*	protein_coding
MALAT1	*SPCS2*	protein_coding
MALAT1	*PLAGL1*	protein_coding
MALAT1	*FBXO30*	protein_coding

lncRNA	*Target*	Gene type
MALAT1	*TNFAIP3*	protein_coding
MALAT1	*CCND2*	protein_coding
MALAT1	*TJP2*	protein_coding
MALAT1	*HEATR1*	protein_coding
MALAT1	*ALG2*	protein_coding
MALAT1	*FCF1*	protein_coding
MALAT1	*NEK9*	protein_coding
MALAT1	*TCP1*	protein_coding
MALAT1	*ETF1*	protein_coding
MALAT1	*TMPO*	protein_coding
MALAT1	*FAM117A*	protein_coding
MALAT1	*LRIF1*	protein_coding
MALAT1	*UBL3*	protein_coding
MALAT1	*ANXA11*	protein_coding
MALAT1	*BBS9*	protein_coding
MALAT1	*HNRNPA2B1*	protein_coding
MALAT1	*POLM*	protein_coding
MALAT1	*SERPINB6*	protein_coding
MALAT1	*CDKN1A*	protein_coding
MALAT1	*NUP153*	protein_coding
MALAT1	*SEPT6*	protein_coding
MALAT1	*PPP1R12C*	protein_coding
MALAT1	*CAPNS1*	protein_coding
MALAT1	*COX6B1*	protein_coding
MALAT1	*IFI6*	protein_coding
MALAT1	*PLEKHG3*	protein_coding
MALAT1	*EMC1*	protein_coding
MALAT1	*EPS15L1*	protein_coding
MALAT1	*CHTF18*	protein_coding
MALAT1	*TUBBP1*	transcribed_processed_pseudogene
MALAT1	*TUBA4A*	protein_coding
MALAT1	*CASD1*	protein_coding
MALAT1	*PRKRIP1*	protein_coding
MALAT1	*SNRPN*	protein_coding
MALAT1	*VPS13C*	protein_coding
MALAT1	*COPB1*	protein_coding
MALAT1	*CCNT1*	protein_coding
MALAT1	*ILF3*	protein_coding
MALAT1	*SLC44A2*	protein_coding
MALAT1	*NEDD8*	protein_coding
MALAT1	*ASH2L*	protein_coding
MALAT1	*TOMM40*	protein_coding
MALAT1	*PXDN*	protein_coding
MALAT1	*UBE2M*	protein_coding
MALAT1	*GGT7*	protein_coding
MALAT1	*PPT1*	protein_coding
MALAT1	*VPS25*	protein_coding

lncRNA	*Target*	Gene type
MALAT1	*TNS4*	protein_coding
MALAT1	*EFR3A*	protein_coding
MALAT1	*UBE2G1*	protein_coding
MALAT1	*ANKRD17*	protein_coding
MALAT1	*EIF5A*	protein_coding
MALAT1	*FLOT2*	protein_coding
MALAT1	*KHDC4*	protein_coding
MALAT1	*DCTN4*	protein_coding
MALAT1	*SLC41A1*	protein_coding
MALAT1	*TPT1*	protein_coding
MALAT1	*WDR74*	protein_coding
MALAT1	*MBD2*	protein_coding
MALAT1	*BHLHE40*	protein_coding
MALAT1	*LDHA*	protein_coding
MALAT1	*ECHDC3*	protein_coding
MALAT1	*CCNH*	protein_coding
MALAT1	*GNL2*	protein_coding
MALAT1	*SLC43A3*	protein_coding
MALAT1	*TAOK3*	protein_coding
MALAT1	*MDFIC*	protein_coding
MALAT1	*ESPL1*	protein_coding
MALAT1	*KRT7*	protein_coding
MALAT1	*SMYD5*	protein_coding
MALAT1	*KCNK1*	protein_coding
MALAT1	*URB2*	protein_coding
MALAT1	*GLUL*	protein_coding
MALAT1	*MRPL44*	protein_coding
MALAT1	*LRCH1*	protein_coding
MALAT1	*LCP1*	protein_coding
MALAT1	*KAT7*	protein_coding
MALAT1	*SAP130*	protein_coding
MALAT1	*DNAJB5*	protein_coding
MALAT1	*DCTN3*	protein_coding
MALAT1	*TJAP1*	protein_coding
MALAT1	*HMGA1*	protein_coding
MALAT1	*FXYD6*	protein_coding
MALAT1	*RTF1*	protein_coding
MALAT1	*PPIG*	protein_coding
MALAT1	*SEPT11*	protein_coding
MALAT1	*FRAS1*	protein_coding
MALAT1	*GABARAPL1*	protein_coding
MALAT1	*WARS*	protein_coding
MALAT1	*SLC12A6*	protein_coding
MALAT1	*SERF2*	protein_coding
MALAT1	*ETFA*	protein_coding
MALAT1	*ARHGAP17*	protein_coding
MALAT1	*UTP4*	protein_coding

lncRNA	*Target*	Gene type
MALAT1	*PCTP*	protein_coding
MALAT1	*G6PC3*	protein_coding
MALAT1	*RMC1*	protein_coding
MALAT1	*HSPG2*	protein_coding
MALAT1	*PTPRF*	protein_coding
MALAT1	*POU2F1*	protein_coding
MALAT1	*SDHC*	protein_coding
MALAT1	*MCL1*	protein_coding
MALAT1	*ANP32E*	protein_coding
MALAT1	*SEMA6C*	protein_coding
MALAT1	*C1orf43*	protein_coding
MALAT1	*RPL32*	protein_coding
MALAT1	*ARL6IP5*	protein_coding
MALAT1	*LIMD1*	protein_coding
MALAT1	*ATG3*	protein_coding
MALAT1	*MUC4*	protein_coding
MALAT1	*KLHL8*	protein_coding
MALAT1	*PHIP*	protein_coding
MALAT1	*DYNLT1*	protein_coding
MALAT1	*NAPRT*	protein_coding
MALAT1	*GTF3C5*	protein_coding
MALAT1	*EIF4EBP2*	protein_coding
MALAT1	*CNNM2*	protein_coding
MALAT1	*PAK1*	protein_coding
MALAT1	*RPS3*	protein_coding
MALAT1	*NCAM1*	protein_coding
MALAT1	*B3GAT3*	protein_coding
MALAT1	*ITGB1*	protein_coding
MALAT1	*PDCD4*	protein_coding
MALAT1	*UBC*	protein_coding
MALAT1	*EXT2*	protein_coding
MALAT1	*NEK7*	protein_coding
MALAT1	*GFRA1*	protein_coding
MALAT1	*BAG3*	protein_coding
MALAT1	*HOMER1*	protein_coding
MALAT1	*IMPACT*	protein_coding
MALAT1	*CC2D1B*	protein_coding
MALAT1	*PPP4R1*	protein_coding
MALAT1	*VOPP1*	protein_coding
MALAT1	*PPARGC1B*	protein_coding
MALAT1	*C8orf37*	protein_coding
MALAT1	*BACH1*	protein_coding
MALAT1	*RPL30*	protein_coding
MALAT1	*TYSND1*	protein_coding
MALAT1	*PRR14*	protein_coding
MALAT1	*RPUSD3*	protein_coding
MALAT1	*RNF111*	protein_coding

lncRNA	*Target*	Gene type
MALAT1	*MYO1E*	protein_coding
MALAT1	*TSC22D3*	protein_coding
MALAT1	*TAB3*	protein_coding
MALAT1	*FMNL2*	protein_coding
MALAT1	*BABAM2*	protein_coding
MALAT1	*CNNM4*	protein_coding
MALAT1	*WASF2*	protein_coding
MALAT1	*TAGLN2*	protein_coding
MALAT1	*ELK4*	protein_coding
MALAT1	*DUSP23*	protein_coding
MALAT1	*F11R*	protein_coding
MALAT1	*MPZ*	protein_coding
MALAT1	*EPB41*	protein_coding
MALAT1	*IGF2BP1*	protein_coding
MALAT1	*CHAF1B*	protein_coding
MALAT1	*ADIPOR1*	protein_coding
MALAT1	*BTG2*	protein_coding
MALAT1	*ADGRG5*	protein_coding
MALAT1	*ABR*	protein_coding
MALAT1	*PRMT2*	protein_coding
MALAT1	*SHC1*	protein_coding
MALAT1	*SLC25A44*	protein_coding
MALAT1	*BDH1*	protein_coding
MALAT1	*SRSF2*	protein_coding
MALAT1	*EMC10*	protein_coding
MALAT1	*SNRNP25*	protein_coding
MALAT1	*LAPTM5*	protein_coding
MALAT1	*IGSF8*	protein_coding
MALAT1	*WDR26*	protein_coding
MALAT1	*SGCB*	protein_coding
MALAT1	*LRRC58*	protein_coding
MALAT1	*RPL22L1*	protein_coding
MALAT1	*RPL9*	protein_coding
MALAT1	*U2SURP*	protein_coding
MALAT1	*TKT*	protein_coding
MALAT1	*TMEM184C*	protein_coding
MALAT1	*MIOS*	protein_coding
MALAT1	*YWHAZ*	protein_coding
MALAT1	*INTS8*	protein_coding
MALAT1	*HNRNPK*	protein_coding
MALAT1	*ARHGAP12*	protein_coding
MALAT1	*REEP3*	protein_coding
MALAT1	*ATP5F1C*	protein_coding
MALAT1	*PRPF18*	protein_coding
MALAT1	*PDZD8*	protein_coding
MALAT1	*TAF1D*	protein_coding
MALAT1	*NDUFB8*	protein_coding

lncRNA	*Target*	Gene type
MALAT1	*PCBD1*	protein_coding
MALAT1	*ZNF202*	protein_coding
MALAT1	*ANAPC16*	protein_coding
MALAT1	*PRTG*	protein_coding
MALAT1	*B2M*	protein_coding
MALAT1	*PCLAF*	protein_coding
MALAT1	*VPS39*	protein_coding
MALAT1	*MARS*	protein_coding
MALAT1	*FBXO22*	protein_coding
MALAT1	*CDK12*	protein_coding
MALAT1	*RPL13*	protein_coding
MALAT1	*TUBA1C*	protein_coding
MALAT1	*LENG8*	protein_coding
MALAT1	*EEF2*	protein_coding
MALAT1	*CYB5D2*	protein_coding
MALAT1	*C19orf48*	protein_coding
MALAT1	*NDUFV1*	protein_coding
MALAT1	*ZNF232*	protein_coding
MALAT1	*SDHAF2*	protein_coding
MALAT1	*RPSA*	protein_coding
MALAT1	*SEPT2*	protein_coding
MALAT1	*PKIG*	protein_coding
MALAT1	*MAT2A*	protein_coding
MALAT1	*INPP5D*	protein_coding
MALAT1	*SLC25A6*	protein_coding
MALAT1	*MMGT1*	protein_coding
MALAT1	*HINT1*	protein_coding
MALAT1	*HIC2*	protein_coding
MALAT1	*LINGO1*	protein_coding
MALAT1	*ALCAM*	protein_coding
MALAT1	*PWWP2A*	protein_coding
MALAT1	*ZNF282*	protein_coding
MALAT1	*LGALS9B*	protein_coding
MALAT1	*UBB*	protein_coding
MALAT1	*HSPA4*	protein_coding
MALAT1	*GSTA4*	protein_coding
MALAT1	*BPTF*	protein_coding
MALAT1	*ZIK1*	protein_coding
MALAT1	*ANO5*	protein_coding
MALAT1	*BSG*	protein_coding
MALAT1	*HCFC1*	protein_coding
MALAT1	*CORO1B*	protein_coding
MALAT1	*CFL1*	protein_coding
MALAT1	*NOC3L*	protein_coding
MALAT1	*RAPH1*	protein_coding
MALAT1	*GPR137*	protein_coding
MALAT1	*RNF26*	protein_coding

lncRNA	*Target*	Gene type
MALAT1	*UQCRH*	protein_coding
MALAT1	*RNF213*	protein_coding
MALAT1	*DPY19L1*	protein_coding
MALAT1	*PRPF8*	protein_coding
MALAT1	*RALGAPA1*	protein_coding
MALAT1	*IGDCC3*	protein_coding
MALAT1	*PTDSS2*	protein_coding
MALAT1	*SMAD2*	protein_coding
MALAT1	*RPL7AP66*	processed_pseudogene
MALAT1	*PLEKHF2*	protein_coding
MALAT1	*LRRN1*	protein_coding
MALAT1	*NFATC2IP*	protein_coding
MALAT1	*TALDO1*	protein_coding
MALAT1	*MAN1B1*	protein_coding
MALAT1	*RPL13AP3*	transcribed_processed_pseudogene
MALAT1	*ZFAS1*	antisense
MALAT1	*RPLP2*	protein_coding
MALAT1	*HNRNPA0*	protein_coding
MALAT1	*ZNF354B*	protein_coding
MALAT1	*EPM2AIP1*	protein_coding
MALAT1	*TMEM107*	protein_coding
MALAT1	*CALR*	protein_coding
MALAT1	*PTPN11*	protein_coding
MALAT1	*MROH1*	protein_coding
MALAT1	*R3HDM2*	protein_coding
MALAT1	*NRXN1*	protein_coding
MALAT1	*ARHGAP45*	protein_coding
MALAT1	*EIF2S3B*	protein_coding
MALAT1	*FRAT2*	protein_coding
MALAT1	*ZNF678*	protein_coding
MALAT1	*C5orf24*	protein_coding
MALAT1	*ADO*	protein_coding
MALAT1	*PNMA8A*	protein_coding
MALAT1	*TMEM259*	protein_coding
MALAT1	*NGRN*	protein_coding
MALAT1	*PYCR1*	protein_coding
MALAT1	*SF3A3*	protein_coding
MALAT1	*TMEM50A*	protein_coding
MALAT1	*TOB2*	protein_coding
MALAT1	*ACTG1*	protein_coding
MALAT1	*BRD7P2*	processed_pseudogene
MALAT1	*NIPSNAP1*	protein_coding
MALAT1	*HDDC3*	protein_coding
MALAT1	*DGAT1*	protein_coding
MALAT1	*RAB11B*	protein_coding
MALAT1	*MRPL40*	protein_coding
MALAT1	*ANKRD46*	protein_coding

lncRNA	*Target*	Gene type
MALAT1	*RPS23*	protein_coding
MALAT1	*SELL*	protein_coding
MALAT1	*ACTBP11*	processed_pseudogene
MALAT1	*NDOR1*	protein_coding
MALAT1	*CLN3*	protein_coding
MALAT1	*SUMO2*	protein_coding
MALAT1	*LITAF*	protein_coding
MALAT1	*JPT1*	protein_coding
MALAT1	*RNU1-75P*	snRNA
MALAT1	*EEF1A1P5*	processed_pseudogene
MALAT1	*NIF3L1*	protein_coding
MALAT1	*IARS*	protein_coding
MALAT1	*LONP1*	protein_coding
MALAT1	*TRRAP*	protein_coding
MALAT1	*PTPN1*	protein_coding
MALAT1	*EPHB4*	protein_coding
MALAT1	*XRCC6*	protein_coding
MALAT1	*NACA*	protein_coding
MALAT1	*SULF2*	protein_coding
MALAT1	*TOMM7*	protein_coding
MALAT1	*MVB12B*	protein_coding
MALAT1	*ARHGEF12*	protein_coding
MALAT1	*FLNA*	protein_coding
MALAT1	*NOP9*	protein_coding
MALAT1	*METTL9*	protein_coding
MALAT1	*DDI2*	protein_coding
MALAT1	*SIPA1L1*	protein_coding
MALAT1	*RPS26*	protein_coding
MALAT1	*OCLN*	protein_coding
MALAT1	*PLCG2*	protein_coding
MALAT1	*RPL12*	protein_coding
MALAT1	*MPZL1*	protein_coding
MALAT1	*SLC29A3*	protein_coding
MALAT1	*MGEA5*	protein_coding
MALAT1	*CTNND1*	protein_coding
MALAT1	*MTCO3P12*	unprocessed_pseudogene
MALAT1	*MT-CO1*	protein_coding
MALAT1	*UBE2J1*	protein_coding
MALAT1	*MTND4LP30*	processed_pseudogene
MALAT1	*MT-ND4*	protein_coding
MALAT1	*MT-ND1*	protein_coding
MALAT1	*PRC1*	protein_coding
MALAT1	*ATG9A*	protein_coding
MALAT1	*APRT*	protein_coding
MALAT1	*MT-CO3*	protein_coding
MALAT1	*RNA5S12*	rRNA
MALAT1	*RNA5S11*	rRNA

lncRNA	*Target*	Gene type
MALAT1	*RNU5E-1*	snRNA
MALAT1	*RNA5S1*	rRNA
MALAT1	*RNU1-150P*	snRNA
MALAT1	*RNA5SP370*	rRNA
MALAT1	*RNU1-94P*	snRNA
MALAT1	*RF00019*	misc_RNA
MALAT1	*RNU5A-1*	snRNA
MALAT1	*RNU1-14P*	snRNA
MALAT1	*RNU1-35P*	snRNA
MALAT1	*RNU1-72P*	snRNA
MALAT1	*RN7SKP131*	misc_RNA
MALAT1	*RNA5S10*	rRNA
MALAT1	*RNU1-18P*	snRNA
MALAT1	*VTRNA1-1*	misc_RNA
MALAT1	*RNU5B-1*	snRNA
MALAT1	*RNA5SP350*	rRNA
MALAT1	*RNU1-21P*	snRNA
MALAT1	*SNORD118*	snoRNA
MALAT1	*RNU1-87P*	snRNA
MALAT1	*RNA5S6*	rRNA
MALAT1	*RN7SKP160*	misc_RNA
MALAT1	*RNU1-124P*	snRNA
MALAT1	*RN7SKP180*	misc_RNA
MALAT1	*RNU4-1*	snRNA
MALAT1	*RNU1-32P*	snRNA
MALAT1	*RNA5SP48*	rRNA
MALAT1	*RNU1-42P*	snRNA
MALAT1	*RN7SKP87*	misc_RNA
MALAT1	*RNU5A-8P*	snRNA
MALAT1	*RNU1-7P*	snRNA
MALAT1	*Y_RNA*	misc_RNA
MALAT1	*RNU6-776P*	snRNA
MALAT1	*RNA5S14*	rRNA
MALAT1	*RNA5SP141*	rRNA
MALAT1	*RNU1-141P*	snRNA
MALAT1	*RNVU1-6*	snRNA
MALAT1	*RNU1-133P*	snRNA
MALAT1	*RNA5SP442*	rRNA
MALAT1	*RNA5SP19*	rRNA
MALAT1	*RN7SKP80*	misc_RNA
MALAT1	*RNVU1-11*	snRNA
MALAT1	*RNU6-1175P*	snRNA
MALAT1	*RNA5SP329*	rRNA
MALAT1	*RNU1-51P*	snRNA
MALAT1	*SNORD14C*	snoRNA
MALAT1	*RNA5SP77*	rRNA
MALAT1	*RNU1-84P*	snRNA

lncRNA	*Target*	Gene type
MALAT1	*RNY3*	misc_RNA
MALAT1	*RNA5SP283*	rRNA
MALAT1	*RNA5S7*	rRNA
MALAT1	*RNU4-2*	snRNA
MALAT1	*HIST2H3A*	protein_coding
MALAT1	*HLA-DMA*	protein_coding
MALAT1	*HLA-DRA*	protein_coding
MALAT1	*BAG6*	protein_coding
MALAT1	*HLA-E*	protein_coding
MALAT1	*HLA-J*	transcribed_unprocessed_pseudogene
MALAT1	*RACK1*	protein_coding
MALAT1	*TMSB4X*	protein_coding
MALAT1	*ITPRIPL2*	protein_coding
MALAT1	*HCP5*	sense_overlapping
MALAT1	*RAB12*	protein_coding
MALAT1	*HLA-A*	protein_coding
MALAT1	*RNVU1-7*	snRNA
MALAT1	*RNU1-28P*	snRNA
MALAT1	*RNU1-39P*	snRNA
MALAT1	*RNU1-1*	snRNA
MALAT1	*RNU1-11P*	snRNA
MALAT1	*RNU1-138P*	snRNA
MALAT1	*RNU1-74P*	snRNA
MALAT1	*RNU6-48P*	snRNA
MALAT1	*RNU1-136P*	snRNA
MALAT1	*RF00409*	snoRNA
MALAT1	*RNU6-5P*	snRNA
MALAT1	*RNU1-2*	snRNA
MALAT1	*RNU1-106P*	snRNA
MALAT1	*RNU6-16P*	snRNA
MALAT1	*SNORD14D*	snoRNA
MALAT1	*RNU1-67P*	snRNA
MALAT1	*RNU1-44P*	snRNA
MALAT1	*RNU1-148P*	snRNA
MALAT1	*RNU6-393P*	snRNA
MALAT1	*RNVU1-15*	snRNA
MALAT1	*RNU1-89P*	snRNA
MALAT1	*RNU1-69P*	snRNA
MALAT1	*RNVU1-1*	snRNA
MALAT1	*RNVU1-17*	snRNA
MALAT1	*RNU1-4*	snRNA
MALAT1	*RNU6ATAC4P*	snRNA
MALAT1	*SNORA40*	snoRNA
MALAT1	*IGKC*	IG_C_gene
MALAT1	*IGKJ5*	IG_J_gene
MALAT1	*IGHG4*	IG_C_gene
MALAT1	*IGHG1*	IG_C_gene

lncRNA	*Target*	Gene type
MALAT1	*RNU1-82P*	snRNA
MALAT1	*RNU1-77P*	snRNA
MALAT1	*RNA5SP86*	rRNA
MALAT1	*RNU1-56P*	snRNA
MALAT1	*NUP62*	protein_coding
MALAT1	*MLLT11*	protein_coding
MALAT1	*NPM1P50*	transcribed_processed_pseudogene
MALAT1	*MXD3*	protein_coding
MALAT1	*RPS3P6*	processed_pseudogene
MALAT1	*PTPRCAP*	protein_coding
MALAT1	*RPS23P2*	processed_pseudogene
MALAT1	*AC026826.1*	processed_pseudogene
MALAT1	*LDHAP5*	processed_pseudogene
MALAT1	*GAPDHP25*	processed_pseudogene
MALAT1	*RPSAP54*	processed_pseudogene
MALAT1	*SLC25A5P3*	processed_pseudogene
MALAT1	*DNASE1*	protein_coding
MALAT1	*EEF1A1P12*	processed_pseudogene
MALAT1	*AC124312.1*	protein_coding
MALAT1	*ALDOAP2*	processed_pseudogene
MALAT1	*TUBAP2*	processed_pseudogene
MALAT1	*ZBED1*	protein_coding
MALAT1	*LINC01588*	lincRNA
MALAT1	*FDPSP5*	processed_pseudogene
MALAT1	*EEF1A1P29*	processed_pseudogene
MALAT1	*RSC1A1*	protein_coding
MALAT1	*GAPDHP72*	transcribed_processed_pseudogene
MALAT1	*AL627402.1*	processed_pseudogene
MALAT1	*AC016739.1*	processed_pseudogene
MALAT1	*RPS18P9*	processed_pseudogene
MALAT1	*RNU6ATAC*	snRNA
MALAT1	*PLXNA4*	protein_coding
MALAT1	*OR2A25*	protein_coding
MALAT1	*SLC12A8*	protein_coding
MALAT1	*UBA52*	protein_coding
MALAT1	*POTEJ*	protein_coding
MALAT1	*RN7SKP111*	misc_RNA
MALAT1	*RNU2-2P*	snRNA
MALAT1	*RNU2-5P*	snRNA
MALAT1	*RNU2-23P*	snRNA
MALAT1	*RNU2-48P*	snRNA
MALAT1	*RNU2-42P*	snRNA
MALAT1	*RNU2-38P*	snRNA
MALAT1	*RNU2-32P*	snRNA
MALAT1	*NUTM2A-AS1*	antisense
MALAT1	*EEF1A1P8*	processed_pseudogene
MALAT1	*EEF1A1P24*	processed_pseudogene

lncRNA	*Target*	Gene type
MALAT1	*AL513328.1*	processed_pseudogene
MALAT1	*AC098614.2*	processed_pseudogene
MALAT1	*AL590762.3*	processed_pseudogene
MALAT1	*HSP90AA2P*	processed_pseudogene
MALAT1	*AC017035.1*	processed_pseudogene
MALAT1	*RPL36P16*	processed_pseudogene
MALAT1	*GAPDHP46*	processed_pseudogene
MALAT1	*PLEKHM1*	protein_coding
MALAT1	*RPS12P26*	transcribed_processed_pseudogene
MALAT1	*AC246787.1*	processed_pseudogene
MALAT1	*NUTM2B-AS1*	antisense
MALAT1	*MTND2P28*	unprocessed_pseudogene
MALAT1	*BX890604.2*	processed_pseudogene
MALAT1	*MTND1P23*	unprocessed_pseudogene
MALAT1	*AL031727.1*	processed_pseudogene
MALAT1	*AC108161.1*	processed_pseudogene
MALAT1	*NOTCH2P1*	processed_pseudogene
MALAT1	*SCDP1*	processed_pseudogene
MALAT1	*FTLP3*	processed_pseudogene
MALAT1	*DANCR*	processed_transcript
MALAT1	*AC005912.1*	processed_pseudogene
MALAT1	*TP73-AS1*	transcribed_unitary_pseudogene
MALAT1	*AC107890.1*	processed_pseudogene
MALAT1	*RPS3AP37*	processed_pseudogene
MALAT1	*AL390728.4*	transcribed_unprocessed_pseudogene
MALAT1	*DHX9P1*	processed_pseudogene
MALAT1	*ATF4P3*	processed_pseudogene
MALAT1	*BX284668.2*	lincRNA
MALAT1	*AL359918.1*	processed_pseudogene
MALAT1	*MTCO1P3*	processed_pseudogene
MALAT1	*MTCO2P12*	unprocessed_pseudogene
MALAT1	*MYL8P*	processed_pseudogene
MALAT1	*MTATP8P2*	processed_pseudogene
MALAT1	*RPL4P4*	processed_pseudogene
MALAT1	*AC245047.2*	processed_pseudogene
MALAT1	*XIST*	lincRNA
MALAT1	*ACBD6*	protein_coding
MALAT1	*AC009245.1*	processed_pseudogene
MALAT1	*DYNLL1P7*	processed_pseudogene
MALAT1	*RPS23P8*	processed_pseudogene
MALAT1	*AC241584.1*	processed_pseudogene
MALAT1	*RPL9P8*	pseudogene
MALAT1	*MIR205HG*	processed_transcript
MALAT1	*RPS18*	protein_coding
MALAT1	*MTND4LP1*	processed_pseudogene
MALAT1	*RPL32P28*	processed_pseudogene
MALAT1	*MTND1P32*	processed_pseudogene

lncRNA	*Target*	Gene type
MALAT1	*AL390728.5*	lincRNA
MALAT1	*RPL3P4*	processed_pseudogene
MALAT1	*RPS11P5*	processed_pseudogene
MALAT1	*EEF1A1P14*	processed_pseudogene
MALAT1	*AC009362.1*	processed_pseudogene
MALAT1	*HOTAIRM1*	antisense
MALAT1	*EEF1A1P6*	processed_pseudogene
MALAT1	*ADH5P4*	processed_pseudogene
MALAT1	*AP000936.3*	processed_pseudogene
MALAT1	*RPL13AP20*	processed_pseudogene
MALAT1	*JRK*	protein_coding
MALAT1	*YWHAEP5*	processed_pseudogene
MALAT1	*GAS5*	processed_transcript
MALAT1	*HLA-B*	protein_coding
MALAT1	*EEF1GP5*	processed_pseudogene
MALAT1	*RPS3AP6*	processed_pseudogene
MALAT1	*AC026784.1*	transcribed_processed_pseudogene
MALAT1	*BTG3P1*	processed_pseudogene
MALAT1	*RPS2P7*	processed_pseudogene
MALAT1	*LINC01553*	lincRNA
MALAT1	*LDHAP3*	processed_pseudogene
MALAT1	*RPL5P9*	processed_pseudogene
MALAT1	*RPL13AP5*	processed_pseudogene
MALAT1	*AC046176.1*	processed_pseudogene
MALAT1	*RPL30P4*	processed_pseudogene
MALAT1	*AC078991.1*	processed_pseudogene
MALAT1	*MTCO1P12*	unprocessed_pseudogene
MALAT1	*ACTG1P19*	processed_pseudogene
MALAT1	*LINC00426*	lincRNA
MALAT1	*RNU1-88P*	snRNA
MALAT1	*SCARNA7*	snoRNA
MALAT1	*RNU1-131P*	snRNA
MALAT1	*RNU1-80P*	snRNA
MALAT1	*SNORD13*	snoRNA
MALAT1	*TXNDC5*	protein_coding
MALAT1	*AC011979.1*	processed_pseudogene
MALAT1	*RPL32P34*	processed_pseudogene
MALAT1	*RN7SL573P*	misc_RNA
MALAT1	*AC073610.1*	processed_pseudogene
MALAT1	*IGKV3-20*	IG_V_gene
MALAT1	*AC104563.1*	processed_pseudogene
MALAT1	*RPSAP12*	processed_pseudogene
MALAT1	*AL132838.1*	processed_pseudogene
MALAT1	*AC132825.1*	unprocessed_pseudogene
MALAT1	*PPIL3*	protein_coding
MALAT1	*RPS12P28*	processed_pseudogene
MALAT1	*AL645465.1*	antisense

lncRNA	*Target*	Gene type
MALAT1	*RN7SL617P*	misc_RNA
MALAT1	*RN7SL70P*	misc_RNA
MALAT1	*AL136126.1*	processed_pseudogene
MALAT1	*CRCP*	protein_coding
MALAT1	*IGKV3-11*	IG_V_gene
MALAT1	*ATP5PO*	protein_coding
MALAT1	*RPL32P26*	processed_pseudogene
MALAT1	*PWP2*	protein_coding
MALAT1	*RN7SL566P*	misc_RNA
MALAT1	*RPL11P5*	processed_pseudogene
MALAT1	*AC108725.1*	processed_pseudogene
MALAT1	*AC100771.1*	processed_pseudogene
MALAT1	*RN7SL575P*	misc_RNA
MALAT1	*MRPL33*	protein_coding
MALAT1	*RPL30P14*	processed_pseudogene
MALAT1	*AC090543.2*	processed_pseudogene
MALAT1	*RPL5P30*	processed_pseudogene
MALAT1	*RPLP0P2*	transcribed_processed_pseudogene
MALAT1	*AC090589.1*	processed_pseudogene
MALAT1	*RPL5P14*	processed_pseudogene
MALAT1	*MRPS6*	protein_coding
MALAT1	*RPS6P22*	processed_pseudogene
MALAT1	*AC116533.1*	processed_pseudogene
MALAT1	*RPS26P43*	processed_pseudogene
MALAT1	*EEF1A1P4*	processed_pseudogene
MALAT1	*MTND4P12*	processed_pseudogene
MALAT1	*MTATP6P1*	unprocessed_pseudogene
MALAT1	*C5orf17*	lincRNA
MALAT1	*BCLAF1P1*	processed_pseudogene
MALAT1	*EEF1A1P9*	processed_pseudogene
MALAT1	*GAPDHP70*	processed_pseudogene
MALAT1	*AC093809.1*	processed_pseudogene
MALAT1	*CHCHD2P7*	processed_pseudogene
MALAT1	*AC068580.4*	protein_coding
MALAT1	*MTCYBP35*	processed_pseudogene
MALAT1	*SEMA3F*	protein_coding
MALAT1	*RBM5*	protein_coding
MALAT1	*SOX8*	protein_coding
MALAT1	*ITGA3*	protein_coding
MALAT1	*YBX2*	protein_coding
MALAT1	*PHTF2*	protein_coding
MALAT1	*ADIPOR2*	protein_coding
MALAT1	*PAFAH1B1*	protein_coding
MALAT1	*TEAD3*	protein_coding
MALAT1	*RPS20*	protein_coding
MALAT1	*UBE3C*	protein_coding
MALAT1	*MATR3*	protein_coding

lncRNA	*Target*	Gene type
MALAT1	*RUFY3*	protein_coding
MALAT1	*IKZF2*	protein_coding
MALAT1	*GAB2*	protein_coding
MALAT1	*MRI1*	protein_coding
MALAT1	*JADE2*	protein_coding
MALAT1	*TPR*	protein_coding
MALAT1	*VAMP3*	protein_coding
MALAT1	*SZRD1*	protein_coding
MALAT1	*GINM1*	protein_coding
MALAT1	*MRPL43*	protein_coding
MALAT1	*SEC61A1*	protein_coding
MALAT1	*POLR3E*	protein_coding
MALAT1	*BCAT1*	protein_coding
MALAT1	*CS*	protein_coding
MALAT1	*EIF4B*	protein_coding
MALAT1	*SPEN*	protein_coding
MALAT1	*SLK*	protein_coding
MALAT1	*NUCKS1*	protein_coding
MALAT1	*PABPC1*	protein_coding
MALAT1	*TCOF1*	protein_coding
MALAT1	*CPSF1*	protein_coding
MALAT1	*HSD17B10*	protein_coding
MALAT1	*FBXW11*	protein_coding
MALAT1	*SMARCE1*	protein_coding
MALAT1	*IGF2BP2*	protein_coding
MALAT1	*VDAC1P1*	processed_pseudogene
MALAT1	*CLASP1*	protein_coding
MALAT1	*MGLL*	protein_coding
MALAT1	*MYDGF*	protein_coding
MALAT1	*SLC25A3*	protein_coding
MALAT1	*RAB7A*	protein_coding
MALAT1	*TOP2B*	protein_coding
MALAT1	*TOLLIP*	protein_coding
MALAT1	*CIC*	protein_coding
MALAT1	*PGM1*	protein_coding
MALAT1	*KAT6A*	protein_coding
MALAT1	*APLP2*	protein_coding
MALAT1	*WBP11*	protein_coding
MALAT1	*KIF3C*	protein_coding
MALAT1	*SCAMP1*	protein_coding
MALAT1	*B4GALT1*	protein_coding
MALAT1	*FTL*	protein_coding
MALAT1	*SRRT*	protein_coding
MALAT1	*PSMC5*	protein_coding
MALAT1	*DOCK3*	protein_coding
MALAT1	*ATRN*	protein_coding
MALAT1	*RPLP0*	protein_coding

lncRNA	*Target*	Gene type
MALAT1	*PXN*	protein_coding
MALAT1	*AARS*	protein_coding
MALAT1	*GLG1*	protein_coding
MALAT1	*DTX2*	protein_coding
MALAT1	*JPH4*	protein_coding
MALAT1	*PSMD5*	protein_coding
MALAT1	*HSP90AB1*	protein_coding
MALAT1	*WASHC2A*	protein_coding
MALAT1	*ATP5F1D*	protein_coding
MALAT1	*PCDH11Y*	protein_coding
MALAT1	*POLRMT*	protein_coding
MALAT1	*MKNK2*	protein_coding
MALAT1	*PPIL2*	protein_coding
MALAT1	*EIF3L*	protein_coding
MALAT1	*SNU13*	protein_coding
MALAT1	*AP1B1*	protein_coding
MALAT1	*MYH9*	protein_coding
MALAT1	*NIN*	protein_coding
MALAT1	*HIF1A*	protein_coding
MALAT1	*PSMB5*	protein_coding
MALAT1	*ARHGAP5*	protein_coding
MALAT1	*CHD8*	protein_coding
MALAT1	*TM9SF1*	protein_coding
MALAT1	*SEC23A*	protein_coding
MALAT1	*SLCO4A1*	protein_coding
MALAT1	*POFUT1*	protein_coding
MALAT1	*VAPA*	protein_coding
MALAT1	*METTL4*	protein_coding
MALAT1	*MCTS2P*	processed_pseudogene
MALAT1	*SMARCA1*	protein_coding
MALAT1	*MAGT1*	protein_coding
MALAT1	*SLC25A15*	protein_coding
MALAT1	*DGKH*	protein_coding
MALAT1	*PSMD7*	protein_coding
MALAT1	*MON1B*	protein_coding
MALAT1	*CD276*	protein_coding
MALAT1	*EIF3E*	protein_coding
MALAT1	*TSTA3*	protein_coding
MALAT1	*MAN2B1*	protein_coding
MALAT1	*HNRNPL*	protein_coding
MALAT1	*SARS2*	protein_coding
MALAT1	*RPS16*	protein_coding
MALAT1	*PLIN3*	protein_coding
MALAT1	*RABAC1*	protein_coding
MALAT1	*KDELR1*	protein_coding
MALAT1	*TNPO2*	protein_coding
MALAT1	*GCDH*	protein_coding

lncRNA	*Target*	Gene type
MALAT1	*PMPCB*	protein_coding
MALAT1	*HBP1*	protein_coding
MALAT1	*MTPN*	protein_coding
MALAT1	*TAX1BP1*	protein_coding
MALAT1	*TMEM248*	protein_coding
MALAT1	*TMEM245*	protein_coding
MALAT1	*RAPGEF1*	protein_coding
MALAT1	*NPDC1*	protein_coding
MALAT1	*GATA3*	protein_coding
MALAT1	*CCDC6*	protein_coding
MALAT1	*RPL28*	protein_coding
MALAT1	*PPIF*	protein_coding
MALAT1	*NUFIP2*	protein_coding
MALAT1	*PSMD3*	protein_coding
MALAT1	*SLC25A11*	protein_coding
MALAT1	*SMARCD2*	protein_coding
MALAT1	*UTP6*	protein_coding
MALAT1	*YWHAE*	protein_coding
MALAT1	*EIF4G2*	protein_coding
MALAT1	*MLEC*	protein_coding
MALAT1	*RAD51AP1*	protein_coding
MALAT1	*OAS3*	protein_coding
MALAT1	*GAPDH*	protein_coding
MALAT1	*AL021546.1*	protein_coding
MALAT1	*MCM3*	protein_coding
MALAT1	*C6orf62*	protein_coding
MALAT1	*PPP2R5D*	protein_coding
MALAT1	*HMGCS1*	protein_coding
MALAT1	*HSPA9*	protein_coding
MALAT1	*TARS*	protein_coding
MALAT1	*LNPEP*	protein_coding
MALAT1	*RPL24*	protein_coding
MALAT1	*PLXNA1*	protein_coding
MALAT1	*EIF4G1*	protein_coding
MALAT1	*ACTR3*	protein_coding
MALAT1	*NRBP1*	protein_coding
MALAT1	*STAT1*	protein_coding
MALAT1	*RPL22*	protein_coding
MALAT1	*QSOX1*	protein_coding
MALAT1	*TROVE2*	protein_coding
MALAT1	*RCAN3*	protein_coding
MALAT1	*KMT2A*	protein_coding
MALAT1	*ABCD4*	protein_coding
MALAT1	*RBM25*	protein_coding
MALAT1	*NRDE2*	protein_coding
MALAT1	*RNF170*	protein_coding
MALAT1	*SCPEP1*	protein_coding

lncRNA	*Target*	Gene type
MALAT1	*SRGN*	protein_coding
MALAT1	*RBM19*	protein_coding
MALAT1	*CDK2*	protein_coding
MALAT1	*TUBA1B*	protein_coding
MALAT1	*OBSL1*	protein_coding
MALAT1	*SRSF6*	protein_coding
MALAT1	*TMEM189-UBE2V1*	protein_coding
MALAT1	*RRP36*	protein_coding
MALAT1	*SLC17A3*	protein_coding
MALAT1	*SOX4*	protein_coding
MALAT1	*BBS2*	protein_coding
MALAT1	*ABCC4*	protein_coding
MALAT1	*NT5C*	protein_coding
MALAT1	*RPL23*	protein_coding
MALAT1	*TRIP10*	protein_coding
MALAT1	*SYMPK*	protein_coding
MALAT1	*PSMF1*	protein_coding
MALAT1	*ROMO1*	protein_coding
MALAT1	*LRRC61*	protein_coding
MALAT1	*TICAM1*	protein_coding
MALAT1	*EMC6*	protein_coding
MALAT1	*MGAT3*	protein_coding
MALAT1	*RAC2*	protein_coding
MALAT1	*CALU*	protein_coding
MALAT1	*ARPP19*	protein_coding
MALAT1	*KIF1C*	protein_coding
MALAT1	*BCL2L2*	protein_coding
MALAT1	*DAD1*	protein_coding
MALAT1	*RHBDF2*	protein_coding
MALAT1	*AFDN*	protein_coding
MALAT1	*LSM4*	protein_coding
MALAT1	*JUND*	protein_coding
MALAT1	*PRRC2B*	protein_coding
MALAT1	*UBA1*	protein_coding
MALAT1	*EMC8*	protein_coding
MALAT1	*MRPS25*	protein_coding
MALAT1	*DIAPH1*	protein_coding
MALAT1	*NDFIP1*	protein_coding
MALAT1	*THOC6*	protein_coding
MALAT1	*TIMM10B*	protein_coding
MALAT1	*IMMT*	protein_coding
MALAT1	*PNISR*	protein_coding
MALAT1	*MATN2*	protein_coding
MALAT1	*RTN3*	protein_coding
MALAT1	*MKRN1*	protein_coding
MALAT1	*SBF2*	protein_coding
MALAT1	*ERG28*	protein_coding

lncRNA	*Target*	Gene type
MALAT1	*NOTCH2*	protein_coding
MALAT1	*ARF3*	protein_coding
MALAT1	*YWHAQ*	protein_coding
MALAT1	*NARS*	protein_coding
MALAT1	*DSC2*	protein_coding
MALAT1	*DSC3*	protein_coding
MALAT1	*NREP*	protein_coding
MALAT1	*PSAT1*	protein_coding
MALAT1	*DMTF1*	protein_coding
MALAT1	*ATP5MC2*	protein_coding
MALAT1	*DNAJC14*	protein_coding
MALAT1	*CDK4*	protein_coding
MALAT1	*RPL13AP25*	processed_pseudogene
MALAT1	*COG3*	protein_coding
MALAT1	*KDELR2*	protein_coding
MALAT1	*DDX56*	protein_coding
MALAT1	*DCAF7*	protein_coding
MALAT1	*RPL35*	protein_coding
MALAT1	*MYC*	protein_coding
MALAT1	*TLN1*	protein_coding
MALAT1	*RPS6*	protein_coding
MALAT1	*IRF4*	protein_coding
MALAT1	*NUMA1*	protein_coding
MALAT1	*RPLP1*	protein_coding
MALAT1	*RABGGTB*	protein_coding
MALAT1	*DBT*	protein_coding
MALAT1	*LRPPRC*	protein_coding
MALAT1	*DUSP5*	protein_coding
MALAT1	*ATIC*	protein_coding
MALAT1	*UBL7*	protein_coding
MALAT1	*PPA2*	protein_coding
MALAT1	*HADH*	protein_coding
MALAT1	*LRIG3*	protein_coding
MALAT1	*TMBIM6*	protein_coding
MALAT1	*SORD*	protein_coding
MALAT1	*IGF1R*	protein_coding
MALAT1	*SEC11A*	protein_coding
MALAT1	*RPS2*	protein_coding
MALAT1	*DEF8*	protein_coding
MALAT1	*TOB1*	protein_coding
MALAT1	*CSNK1D*	protein_coding
MALAT1	*BRD4*	protein_coding
MALAT1	*RPS11*	protein_coding
MALAT1	*RPL13A*	protein_coding
MALAT1	*RPL11*	protein_coding
MALAT1	*WDTC1*	protein_coding
MALAT1	*PPOX*	protein_coding

lncRNA	*Target*	Gene type
MALAT1	*HDGF*	protein_coding
MALAT1	*SF3B4*	protein_coding
MALAT1	*PIP5K1A*	protein_coding
MALAT1	*UBAP2L*	protein_coding
MALAT1	*ILF2*	protein_coding
MALAT1	*ARF1*	protein_coding
MALAT1	*CNIH4*	protein_coding
MALAT1	*PDIA6*	protein_coding
MALAT1	*TEX261*	protein_coding
MALAT1	*AMMECR1L*	protein_coding
MALAT1	*FANCD2*	protein_coding
MALAT1	*CNOT9*	protein_coding
MALAT1	*TCTA*	protein_coding
MALAT1	*MARCH6*	protein_coding
MALAT1	*LHFPL2*	protein_coding
MALAT1	*CXCL14*	protein_coding
MALAT1	*PCYOX1L*	protein_coding
MALAT1	*FAM193B*	protein_coding
MALAT1	*ABT1*	protein_coding
MALAT1	*RPL7L1*	protein_coding
MALAT1	*SLC18B1*	protein_coding
MALAT1	*SH3KBP1*	protein_coding
MALAT1	*NDUFB11*	protein_coding
MALAT1	*OGT*	protein_coding
MALAT1	*SIGMAR1*	protein_coding
MALAT1	*CDHR1*	protein_coding
MALAT1	*ADD3*	protein_coding
MALAT1	*RGS10*	protein_coding
MALAT1	*BTBD10*	protein_coding
MALAT1	*ALDOA*	protein_coding
MALAT1	*FREM2*	protein_coding
MALAT1	*ANKRD50*	protein_coding
MALAT1	*DST*	protein_coding
MALAT1	*TMEM123*	protein_coding
MALAT1	*MBNL1*	protein_coding
MALAT1	*HNRNPDL*	protein_coding
MALAT1	*UTRN*	protein_coding
MALAT1	*CARHSP1*	protein_coding
MALAT1	*PTPRD*	protein_coding
MALAT1	*TOMM70*	protein_coding
MALAT1	*UCHL1*	protein_coding
MALAT1	*CCDC144CP*	transcribed_processed_pseudogene
MALAT1	*AGPAT5*	protein_coding
MALAT1	*ZFYVE27*	protein_coding
MALAT1	*SLC16A1*	protein_coding
MALAT1	*LARP1*	protein_coding
MALAT1	*EEF1A1*	protein_coding

lncRNA	*Target*	Gene type
MALAT1	*UBE2L6*	protein_coding
MALAT1	*UTP14A*	protein_coding
MALAT1	*EIF4A2*	protein_coding
MALAT1	*SMG1*	protein_coding
MALAT1	*CPT2*	protein_coding
MALAT1	*CCNB2*	protein_coding
MALAT1	*RER1*	protein_coding
MALAT1	*C21orf59*	protein_coding
MALAT1	*LAD1*	protein_coding
MALAT1	*SIM2*	protein_coding
MALAT1	*RSPRY1*	protein_coding
MALAT1	*CBS*	protein_coding
MALAT1	*CSTB*	protein_coding
MALAT1	*DIP2A*	protein_coding
MALAT1	*ZER1*	protein_coding
MALAT1	*CRTC2*	protein_coding
MALAT1	*ANO10*	protein_coding
MALAT1	*RPL8*	protein_coding
MALAT1	*LSM12*	protein_coding
MALAT1	*LBHD1*	protein_coding
MALAT1	*SLAMF6*	protein_coding
MALAT1	*SCNM1*	protein_coding
MALAT1	*ATP1A1*	protein_coding
MALAT1	*CCT3*	protein_coding
MALAT1	*STT3B*	protein_coding
MALAT1	*SUCLG1*	protein_coding
MALAT1	*SLMAP*	protein_coding
MALAT1	*PRKCD*	protein_coding
MALAT1	*PBRM1*	protein_coding
MALAT1	*CDC25A*	protein_coding
MALAT1	*DCAF13*	protein_coding
MALAT1	*WASHC5*	protein_coding
MALAT1	*METTL2B*	protein_coding
MALAT1	*HGSNAT*	protein_coding
MALAT1	*ARF6*	protein_coding
MALAT1	*HDGFL3*	protein_coding
MALAT1	*MCM7*	protein_coding
MALAT1	*CENPV*	protein_coding
MALAT1	*YWHAB*	protein_coding
MALAT1	*PDIA3*	protein_coding
MALAT1	*NUDT21*	protein_coding
MALAT1	*DOLPP1*	protein_coding
MALAT1	*IGF2*	protein_coding
MALAT1	*ZNF646*	protein_coding
MALAT1	*TUBA1A*	protein_coding
MALAT1	*RCOR2*	protein_coding
MALAT1	*SRP68*	protein_coding

lncRNA	*Target*	Gene type
MALAT1	*SETD5*	protein_coding
MALAT1	*DDIT4*	protein_coding
MALAT1	*FEN1*	protein_coding
MALAT1	*SERINC2*	protein_coding
MALAT1	*PCSK9*	protein_coding
MALAT1	*AL669983.1*	processed_pseudogene
MALAT1	*PTK2*	protein_coding
MALAT1	*RNASE6*	protein_coding
MALAT1	*CLIC4*	protein_coding
MALAT1	*CKAP2L*	protein_coding
MALAT1	*HEXDC*	protein_coding
MALAT1	*TRAPPC1*	protein_coding
MALAT1	*RNF150*	protein_coding
MALAT1	*HOXD4*	protein_coding
MALAT1	*PDCD6IP*	protein_coding
MALAT1	*NFXL1*	protein_coding
MALAT1	*PYM1*	protein_coding
MALAT1	*ELOVL6*	protein_coding
MALAT1	*RASA4B*	protein_coding
MALAT1	*LSM3*	protein_coding
MALAT1	*RPS9*	protein_coding
MALAT1	*ZNF160*	protein_coding
MALAT1	*ATP6V0E2*	protein_coding
MALAT1	*TPPP*	protein_coding
MALAT1	*KRT13*	protein_coding
MALAT1	*KRCC1*	protein_coding
MALAT1	*AHSA2P*	transcribed_unitary_pseudogene
MALAT1	*VEGFB*	protein_coding
MALAT1	*JUP*	protein_coding
MALAT1	*DDX23*	protein_coding
MALAT1	*RPL4*	protein_coding
MALAT1	*RPL15*	protein_coding
MALAT1	*SRP72*	protein_coding
MALAT1	*MARCKSL1*	protein_coding
MALAT1	*RAB6A*	protein_coding
MALAT1	*RPL37AP8*	processed_pseudogene
MALAT1	*SHMT1*	protein_coding
MALAT1	*DEAF1*	protein_coding
MALAT1	*WDR73*	protein_coding
MALAT1	*TGIF1*	protein_coding
MALAT1	*TBL1XR1*	protein_coding
MALAT1	*ZNF518A*	protein_coding
MALAT1	*ZBTB41*	protein_coding
MALAT1	*RIC8A*	protein_coding
MALAT1	*RPL10P16*	processed_pseudogene
MALAT1	*GTPBP6*	protein_coding
MALAT1	*PARP10*	protein_coding

lncRNA	*Target*	Gene type
MALAT1	*COX5A*	protein_coding
MALAT1	*C17orf62*	protein_coding
MALAT1	*TUFM*	protein_coding
MALAT1	*RCC2*	protein_coding
MALAT1	*TIGD5*	protein_coding
MALAT1	*C14orf119*	protein_coding
MALAT1	*FO393411.1*	processed_pseudogene
MALAT1	*SSR4*	protein_coding
MALAT1	*EHMT1*	protein_coding
MALAT1	*MUC16*	protein_coding
MALAT1	*SETD2*	protein_coding
MALAT1	*RNF41*	protein_coding
MALAT1	*MRPS16*	protein_coding
MALAT1	*CACNB4*	protein_coding
MALAT1	*KPNA2*	protein_coding
MALAT1	*TTC3*	protein_coding
MALAT1	*GLUD2*	protein_coding
MALAT1	*AP2A2*	protein_coding
MALAT1	*GPC6*	protein_coding
MALAT1	*SMDT1*	protein_coding
MALAT1	*HSP90AB3P*	processed_pseudogene
MALAT1	*DDX41*	protein_coding
MALAT1	*DAZAP2*	protein_coding
MALAT1	*AC026410.1*	processed_pseudogene
MALAT1	*TSSC4*	protein_coding
MALAT1	*WDR27*	protein_coding
MALAT1	*RBM33*	protein_coding
MALAT1	*HSF1*	protein_coding
MALAT1	*RGPD2*	protein_coding
MALAT1	*HS6ST3*	protein_coding
MALAT1	*NR2F2*	protein_coding
MALAT1	*P4HB*	protein_coding
MALAT1	*ZFP36L1*	protein_coding
MALAT1	*KRT5*	protein_coding
MALAT1	*EDARADD*	protein_coding
MALAT1	*SEPT10*	protein_coding
MALAT1	*SMYD4*	protein_coding
MALAT1	*UBE2H*	protein_coding
MALAT1	*ZFP91*	protein_coding
MALAT1	*PTMA*	protein_coding
MALAT1	*HIST1H1C*	protein_coding
MALAT1	*TUBB4B*	protein_coding
MALAT1	*C19orf54*	protein_coding
MALAT1	*RPL10AP2*	processed_pseudogene
MALAT1	*CLDN4*	protein_coding
MALAT1	*TUBB*	protein_coding
MALAT1	*PPIA*	protein_coding

lncRNA	*Target*	Gene type
MALAT1	*C20orf204*	protein_coding
MALAT1	*SLC6A9*	protein_coding
MALAT1	*PCBP2*	protein_coding
MALAT1	*AC107956.1*	processed_pseudogene
MALAT1	*FAR1*	protein_coding
MALAT1	*PHF2*	protein_coding
MALAT1	*PSAP*	protein_coding
MALAT1	*RPL37A*	protein_coding
MALAT1	*MRPL42*	protein_coding
MALAT1	*MIER1*	protein_coding
MALAT1	*QRICH1*	protein_coding
MALAT1	*UBL5*	protein_coding
MALAT1	*MT-CO2*	protein_coding
MALAT1	*TOGARAM1*	protein_coding
MALAT1	*LDB1*	protein_coding
MALAT1	*MT-ND2*	protein_coding
MALAT1	*MT-ND5*	protein_coding
MALAT1	*ZNF277*	protein_coding
MALAT1	*MT-ATP6*	protein_coding
MALAT1	*RPL39*	protein_coding
MALAT1	*PJA2*	protein_coding
MALAT1	*RNU6-208P*	snRNA
MALAT1	*RN7SKP104*	misc_RNA
MALAT1	*RN7SKP95*	misc_RNA
MALAT1	*RNA5SP426*	rRNA
MALAT1	*RNA5SP358*	rRNA
MALAT1	*RNU5B-2P*	snRNA
MALAT1	*SNORA73B*	snoRNA
MALAT1	*RNU6-984P*	snRNA
MALAT1	*RNU1-19P*	snRNA
MALAT1	*RNA5SP382*	rRNA
MALAT1	*RNA5SP352*	rRNA
MALAT1	*SNORA63*	snoRNA
MALAT1	*RNU1-49P*	snRNA
MALAT1	*RNA5S17*	rRNA
MALAT1	*RNA5S4*	rRNA
MALAT1	*RNA5SP74*	rRNA
MALAT1	*RNA5-8SP2*	rRNA
MALAT1	*RNA5SP429*	rRNA
MALAT1	*SNORA74A*	snoRNA
MALAT1	*RNA5SP336*	rRNA
MALAT1	*RNY1*	misc_RNA
MALAT1	*RNU4-76P*	snRNA
MALAT1	*RNA5S2*	rRNA
MALAT1	*RNU6-7*	snRNA
MALAT1	*RNA5SP267*	rRNA
MALAT1	*RNU1-43P*	snRNA

lncRNA	*Target*	Gene type
MALAT1	*RNU1-100P*	snRNA
MALAT1	*RNA5S16*	rRNA
MALAT1	*RNU6-61P*	snRNA
MALAT1	*RN7SKP187*	misc_RNA
MALAT1	*RNA5S13*	rRNA
MALAT1	*SKIV2L*	protein_coding
MALAT1	*C6orf48*	protein_coding
MALAT1	*LSM2*	protein_coding
MALAT1	*CSNK2B*	protein_coding
MALAT1	*HLA-C*	protein_coding
MALAT1	*GABBR1*	protein_coding
MALAT1	*DCTN1*	protein_coding
MALAT1	*CCL4L1*	protein_coding
MALAT1	*E2F4*	protein_coding
MALAT1	*IPO7*	protein_coding
MALAT1	*MUC19*	protein_coding
MALAT1	*RNU1-27P*	snRNA
MALAT1	*RNU6-25P*	snRNA
MALAT1	*RNU6-672P*	snRNA
MALAT1	*RNVU1-18*	snRNA
MALAT1	*RNU6-116P*	snRNA
MALAT1	*RNU6-42P*	snRNA
MALAT1	*RNU6-4P*	snRNA
MALAT1	*RNU6-31P*	snRNA
MALAT1	*RNU6-905P*	snRNA
MALAT1	*RNA5SP122*	rRNA
MALAT1	*RNU6-18P*	snRNA
MALAT1	*RNU6-140P*	snRNA
MALAT1	*RNVU1-14*	snRNA
MALAT1	*RNU1-3*	snRNA
MALAT1	*SNORA66*	snoRNA
MALAT1	*MT-RNR2*	Mt_rRNA
MALAT1	*STK38L*	protein_coding
MALAT1	*MT-RNR1*	Mt_rRNA
MALAT1	*RF00568*	snoRNA
MALAT1	*MT-ND4L*	protein_coding
MALAT1	*RPL12P38*	transcribed_processed_pseudogene
MALAT1	*EEF1A1P16*	processed_pseudogene
MALAT1	*RPL18AP3*	processed_pseudogene
MALAT1	*TPT1P13*	processed_pseudogene
MALAT1	*RPLP0P6*	processed_pseudogene
MALAT1	*PPP1CB*	protein_coding
MALAT1	*RPL13P2*	processed_pseudogene
MALAT1	*RPL13AP7*	processed_pseudogene
MALAT1	*UBD*	protein_coding
MALAT1	*TAX1BP3*	protein_coding
MALAT1	*PAXIP1-AS2*	antisense

lncRNA	*Target*	Gene type
MALAT1	*RPS15AP1*	processed_pseudogene
MALAT1	*AL354710.1*	processed_pseudogene
MALAT1	*SRSF9P1*	processed_pseudogene
MALAT1	*AC011825.1*	processed_pseudogene
MALAT1	*RPL13P12*	processed_pseudogene
MALAT1	*AP000354.1*	processed_pseudogene
MALAT1	*RPL17-C18orf32*	protein_coding
MALAT1	*IFI30*	protein_coding
MALAT1	*RPL18AP8*	processed_pseudogene
MALAT1	*AL391416.1*	processed_pseudogene
MALAT1	*RPL21P119*	processed_pseudogene
MALAT1	*RNU6-908P*	snRNA
MALAT1	*RN7SKP281*	misc_RNA
MALAT1	*RNU6-628P*	snRNA
MALAT1	*RNA5SP225*	rRNA
MALAT1	*AC023157.1*	processed_pseudogene
MALAT1	*RPS8P10*	unprocessed_pseudogene
MALAT1	*SNHG14*	processed_transcript
MALAT1	*AC211485.1*	processed_pseudogene
MALAT1	*RPL8P2*	processed_pseudogene
MALAT1	*AL138785.1*	processed_pseudogene
MALAT1	*AC005515.1*	transcribed_unprocessed_pseudogene
MALAT1	*UQCRFS1P1*	processed_pseudogene
MALAT1	*GAPDHP73*	processed_pseudogene
MALAT1	*RPS14P4*	processed_pseudogene
MALAT1	*RPS4XP2*	processed_pseudogene
MALAT1	*AC007285.1*	antisense
MALAT1	*AC092035.1*	processed_pseudogene
MALAT1	*FTH1P16*	processed_pseudogene
MALAT1	*RPL3P2*	processed_pseudogene
MALAT1	*TPT1P2*	processed_pseudogene
MALAT1	*PPIAP19*	processed_pseudogene
MALAT1	*AC131235.1*	processed_pseudogene
MALAT1	*TXNP5*	processed_pseudogene
MALAT1	*MT-ATP8*	protein_coding
MALAT1	*AL158201.1*	processed_pseudogene
MALAT1	*FTLP17*	processed_pseudogene
MALAT1	*OST4*	protein_coding
MALAT1	*SNRPD2P1*	processed_pseudogene
MALAT1	*GDI2P1*	processed_pseudogene
MALAT1	*PNPT1P1*	processed_pseudogene
MALAT1	*MTND2P20*	processed_pseudogene
MALAT1	*AL450405.1*	processed_pseudogene
MALAT1	*RPL4P5*	processed_pseudogene
MALAT1	*AC078817.1*	processed_pseudogene
MALAT1	*TCEA1P2*	processed_pseudogene
MALAT1	*AL512488.1*	sense_intronic

lncRNA	*Target*	Gene type
MALAT1	*MTCO1P53*	processed_pseudogene
MALAT1	*ACTG1P10*	processed_pseudogene
MALAT1	*Z97353.1*	processed_pseudogene
MALAT1	*DLEU2*	antisense
MALAT1	*MTND4P24*	processed_pseudogene
MALAT1	*RPL4P2*	processed_pseudogene
MALAT1	*Z74021.1*	processed_pseudogene
MALAT1	*FTLP2*	processed_pseudogene
MALAT1	*ACTBP12*	processed_pseudogene
MALAT1	*BX842559.2*	processed_pseudogene
MALAT1	*AC007969.1*	processed_pseudogene
MALAT1	*RPS15AP11*	processed_pseudogene
MALAT1	*HSPA8P1*	processed_pseudogene
MALAT1	*AL592293.2*	processed_pseudogene
MALAT1	*XRCC6P2*	processed_pseudogene
MALAT1	*AL035456.1*	processed_pseudogene
MALAT1	*RPL10AP5*	processed_pseudogene
MALAT1	*KIAA0040*	protein_coding
MALAT1	*PKMP1*	processed_pseudogene
MALAT1	*AC099654.3*	unprocessed_pseudogene
MALAT1	*AC091685.2*	processed_pseudogene
MALAT1	*AL080243.2*	processed_pseudogene
MALAT1	*SMG1P1*	transcribed_unprocessed_pseudogene
MALAT1	*KIFC1*	protein_coding
MALAT1	*AMD1P2*	processed_pseudogene
MALAT1	*MTCO1P18*	unprocessed_pseudogene
MALAT1	*RNU6-893P*	snRNA
MALAT1	*RNU6-671P*	snRNA
MALAT1	*RN7SL87P*	misc_RNA
MALAT1	*RPL37P2*	processed_pseudogene
MALAT1	*RPS4XP22*	processed_pseudogene
MALAT1	*C1orf226*	protein_coding
MALAT1	*RN7SL674P*	misc_RNA
MALAT1	*RPS4XP13*	processed_pseudogene
MALAT1	*AC010343.1*	processed_pseudogene
MALAT1	*MTATP8P1*	unprocessed_pseudogene
MALAT1	*AP001024.1*	processed_pseudogene
MALAT1	*AC132217.1*	3prime_overlapping_ncRNA
MALAT1	*RN7SL128P*	misc_RNA
MALAT1	*RNA5-8S5*	rRNA
MALAT1	*ARHGAP8*	protein_coding
MALAT1	*PSMC1P1*	processed_pseudogene
MALAT1	*MTCO1P55*	processed_pseudogene
MALAT1	*RPL29P23*	processed_pseudogene
MALAT1	*RPL7AP6*	processed_pseudogene
MALAT1	*AC092597.1*	processed_pseudogene
MALAT1	*AC073861.1*	processed_pseudogene

lncRNA	*Target*	Gene type
MALAT1	*RPL12P32*	processed_pseudogene
MALAT1	*RN7SL685P*	misc_RNA
MALAT1	*HIST1H2APS2*	transcribed_processed_pseudogene
MALAT1	*MICAL3*	protein_coding
MALAT1	*RN7SL861P*	misc_RNA
MALAT1	*AC115223.1*	processed_pseudogene
MALAT1	*STMP1*	protein_coding
MALAT1	*RPS4XP14*	processed_pseudogene
MALAT1	*EEF1A1P10*	processed_pseudogene
MALAT1	*AP000942.1*	processed_pseudogene
MALAT1	*RN7SL610P*	misc_RNA
MALAT1	*TMEM199*	protein_coding
MALAT1	*P2RY11*	protein_coding
MALAT1	*RN7SL151P*	misc_RNA
MALAT1	*AC024293.1*	processed_pseudogene
MALAT1	*ENO1P1*	transcribed_processed_pseudogene
MALAT1	*UBE2V1*	protein_coding
MALAT1	*MTCYBP18*	processed_pseudogene
MALAT1	*NEAT1*	lincRNA
MALAT1	*PRR5-ARHGAP8*	protein_coding
MALAT1	*AC008758.3*	transcribed_unprocessed_pseudogene
MALAT1	*GAPDHP40*	processed_pseudogene
MALAT1	*MTND5P11*	processed_pseudogene
MALAT1	*AC025458.1*	processed_pseudogene
MALAT1	*EEF1A1P19*	processed_pseudogene
MALAT1	*YJEFN3*	protein_coding
MALAT1	*AC104619.3*	processed_pseudogene
MALAT1	*EEF1A1P13*	processed_pseudogene
MALAT1	*GAPDHP62*	processed_pseudogene
MALAT1	*AC024451.1*	unprocessed_pseudogene
MALAT1	*MTND5P12*	processed_pseudogene
MALAT1	*RNA5-8SP6*	rRNA
MALAT1	*RNU6-585P*	snRNA
MALAT1	*RNU6-346P*	snRNA
MALAT1	*RNY3P15*	misc_RNA
MALAT1	*RNA5SP348*	rRNA
MALAT1	*RNU6-1332P*	snRNA
MALAT1	*TRNP1*	protein_coding
MALAT1	*IGHGP*	IG_C_pseudogene
MALAT1	*LINC01933*	lincRNA
MALAT1	*AL136295.1*	protein_coding
MALAT1	*EEF1G*	protein_coding
MALAT1	*AP001646.2*	processed_pseudogene
MALAT1	*ZFP91-CNTF*	protein_coding
MALAT1	*MTCO1P15*	processed_pseudogene
MALAT1	*AP002990.1*	protein_coding
MALAT1	*Z82188.1*	pseudogene

lncRNA	*Target*	Gene type
MALAT1	*MTRNR2L3*	protein_coding
MALAT1	*HSPA8P5*	processed_pseudogene
MALAT1	*AP001888.1*	processed_pseudogene
MALAT1	*AP001453.4*	lincRNA
MALAT1	*P2RX5-TAX1BP3*	protein_coding
MALAT1	*AC091078.2*	processed_pseudogene
MALAT1	*AL355075.4*	antisense
MALAT1	*MTCO1P2*	unprocessed_pseudogene
MALAT1	*MTND5P32*	processed_pseudogene
MALAT1	*AL136295.4*	protein_coding
MALAT1	*AC023813.2*	processed_pseudogene
MALAT1	*ATP5PBP7*	processed_pseudogene
MALAT1	*TUBAP4*	transcribed_processed_pseudogene
MALAT1	*AC108134.2*	lincRNA
MALAT1	*MTCO3P24*	unprocessed_pseudogene
MALAT1	*MTCO1P40*	processed_pseudogene
MALAT1	*AC138761.1*	transcribed_unprocessed_pseudogene
MALAT1	*SNORD3A*	snoRNA
MALAT1	*AC073508.2*	protein_coding
MALAT1	*RN7SL230P*	misc_RNA
MALAT1	*TIMM23*	protein_coding
MALAT1	*RN7SL166P*	misc_RNA
MALAT1	*RPL17*	protein_coding
MALAT1	*RN7SL444P*	misc_RNA
MALAT1	*RASSF5*	protein_coding
MALAT1	*RNA28S5*	rRNA
MALAT1	*AKR1B1P7*	processed_pseudogene
MALAT1	*MTCO2P2*	processed_pseudogene
MALAT1	*ZNF224*	protein_coding
MALAT1	*AC007192.1*	protein_coding
MALAT1	*AC008758.4*	protein_coding
MALAT1	*MTRNR2L12*	protein_coding
MALAT1	*MTRNR2L11*	protein_coding
MALAT1	*MTND4LP5*	processed_pseudogene
MALAT1	*RNA18S5*	rRNA
MALAT1	*POM121C*	protein_coding
MALAT1	*AC093668.2*	protein_coding
MALAT1	*CBSL*	protein_coding
MALAT1	*SYNRG*	protein_coding
MALAT1	*DUSP14*	protein_coding
MALAT1	*UHRF1*	protein_coding
MALAT1	*CCL3L1*	protein_coding
MALAT1	*AC245014.3*	lincRNA
MALAT1	*HIST1H2AE*	protein_coding
MALAT1	*GAPDHP41*	processed_pseudogene
MALAT1	*AC009336.2*	protein_coding
MALAT1	*C11orf98*	protein_coding

lncRNA	*Target*	Gene type
MALAT1	*AC020765.3*	unprocessed_pseudogene
MALAT1	*AC135068.6*	processed_pseudogene
MALAT1	*AD000090.1*	antisense
MALAT1	*hsa-miR-7641*	miRNA
MALAT1	*Met_tRNA*	tRNA
MALAT1	*Asp_tRNA*	tRNA
MALAT1	*Pseudo_tRNA*	tRNA
MALAT1	*His_tRNA*	tRNA
MALAT1	*Arg_tRNA*	tRNA
MALAT1	*Leu_tRNA*	tRNA
MALAT1	*Glu_tRNA*	tRNA
MALAT1	*Ala_tRNA*	tRNA
MALAT1	*Lys_tRNA*	tRNA
MALAT1	*Gly_tRNA*	tRNA
MALAT1	*RNU1-61P*	snRNA
MALAT1	*RNU2-35P*	snRNA
MALAT1	*RNY5*	misc_RNA
MALAT1	*RNU1-135P*	snRNA
MALAT1	*RNU2-46P*	snRNA
MALAT1	*RNU1-117P*	snRNA
MALAT1	*PRKDC*	protein_coding
MALAT1	*AC022861.1*	processed_pseudogene
MALAT1	*TMED10P1*	processed_pseudogene
MALAT1	*MTCYBP41*	processed_pseudogene
MALAT1	*PRR13P2*	processed_pseudogene
MALAT1	*BORCS8*	protein_coding
MALAT1	*AP000781.2*	protein_coding
MALAT1	*DPP3*	protein_coding
MALAT1	*BRK1*	protein_coding
MALAT1	*AC018523.1*	processed_pseudogene
MALAT1	*PGAM1P8*	transcribed_processed_pseudogene
MALAT1	*NPM1P35*	processed_pseudogene
MALAT1	*AL133352.1*	protein_coding
MALAT1	*MTRNR2L8*	protein_coding
MALAT1	*AP000763.2*	processed_pseudogene
MALAT1	*ATF4P4*	transcribed_processed_pseudogene
MALAT1	*SUPT16HP1*	processed_pseudogene
MALAT1	*RPL41P5*	processed_pseudogene
MALAT1	*AC008731.1*	pseudogene
MALAT1	*AL139819.1*	pseudogene
MALAT1	*AP003108.2*	protein_coding
MALAT1	*MTRNR2L1*	protein_coding
MALAT1	*AC140481.3*	pseudogene
MALAT1	*AL021707.2*	pseudogene
MALAT1	*EIF3LP1*	processed_pseudogene
MALAT1	*PPIAP4*	processed_pseudogene
MALAT1	*AC104390.1*	processed_pseudogene

lncRNA	*Target*	Gene type
MALAT1	*BLOC1S5-TXNDC5*	protein_coding
MALAT1	*AC087632.1*	protein_coding
MALAT1	*EEF1A1P22*	processed_pseudogene
MALAT1	*AC091167.2*	protein_coding
MALAT1	*AC007906.1*	sense_intronic
MALAT1	*AC010542.4*	lincRNA
MALAT1	*AC138894.1*	protein_coding
MALAT1	*SNORD3B-2*	snoRNA
MALAT1	*AC010547.6*	unprocessed_pseudogene
MALAT1	*AC007952.4*	lincRNA
MALAT1	*GTF2I*	protein_coding
MALAT1	*MTCO3P13*	unprocessed_pseudogene
MALAT1	*SNORD3C*	snoRNA
MALAT1	*SNORD3B-1*	snoRNA
MALAT1	*AC005899.4*	processed_transcript
MALAT1	*MTCYBP13*	unprocessed_pseudogene
MALAT1	*AP000902.1*	processed_pseudogene
MALAT1	*ACTBP9*	processed_pseudogene
MALAT1	*S1PR2*	protein_coding
MALAT1	*AC011503.2*	sense_intronic
MALAT1	*SNHG8*	lincRNA
MALAT1	*AL356488.2*	lincRNA
MALAT1	*AL360012.1*	lincRNA
MALAT1	*AL591806.3*	protein_coding
MALAT1	*MTCYBP22*	processed_pseudogene
MALAT1	*MTCO3P22*	processed_pseudogene
MALAT1	*MTND4P35*	processed_pseudogene
MALAT1	*MTCO1P22*	processed_pseudogene
MALAT1	*MTND5P10*	processed_pseudogene
MALAT1	*SNORD14A*	snoRNA
MALAT1	*RN7SL828P*	misc_RNA
MALAT1	*RNA5SP506*	rRNA
MALAT1	*AL135925.1*	lincRNA
MALAT1	*AC073610.3*	protein_coding
MALAT1	*SNURF*	protein_coding
MALAT1	*AC019176.2*	unprocessed_pseudogene
MALAT1	*FP565260.1*	protein_coding
MALAT1	*HIST1H3A*	protein_coding
MALAT1	*RIMBP3*	protein_coding
MALAT1	*SRCIN1*	protein_coding
MALAT1	*MARCKS*	protein_coding
MALAT1	*AC090498.1*	processed_pseudogene
MALAT1	*AL022311.1*	sense_overlapping
MALAT1	*SLFNL1-AS1*	antisense
MALAT1	*BLACAT1*	lincRNA
MALAT1	*AL157392.5*	protein_coding
MALAT1	*AL139099.4*	non_coding

lncRNA	*Target*	Gene type
MALAT1	*AC008038.1*	processed_pseudogene
MALAT1	*AC006511.5*	processed_transcript
MALAT1	*AP003175.1*	processed_transcript
MALAT1	*AC068946.2*	protein_coding
MALAT1	*AC068831.7*	protein_coding
MALAT1	*AL358113.1*	protein_coding
MALAT1	*RNA18N5*	rRNA
MALAT1	*hsa-miR-4485*	miRNA
MALAT1	*Thr_tRNA*	tRNA
MALAT1	*Cys_tRNA*	tRNA
TUG1	*SPAST*	protein_coding
TUG1	*ZNF839*	protein_coding
TUG1	*CDH1*	protein_coding
TUG1	*FOXN3*	protein_coding
TUG1	*SLC2A3*	protein_coding
TUG1	*VPS35*	protein_coding
TUG1	*RPL31*	protein_coding
TUG1	*TRIP13*	protein_coding
TUG1	*SLC1A3*	protein_coding
TUG1	*STK17B*	protein_coding
TUG1	*ITPKC*	protein_coding
TUG1	*ago/01*	protein_coding
TUG1	*MSH2*	protein_coding
TUG1	*ATP5F1D*	protein_coding
TUG1	*RANBP1*	protein_coding
TUG1	*SNRPD3*	protein_coding
TUG1	*GTPBP1*	protein_coding
TUG1	*ADNP*	protein_coding
TUG1	*HAS3*	protein_coding
TUG1	*NOMO3*	protein_coding
TUG1	*TTC23*	protein_coding
TUG1	*TUBB4A*	protein_coding
TUG1	*HSPA8*	protein_coding
TUG1	*PTGES3*	protein_coding
TUG1	*SUDS3*	protein_coding
TUG1	*SRSF3*	protein_coding
TUG1	*MDGA1*	protein_coding
TUG1	*SRF*	protein_coding
TUG1	*TMCO6*	protein_coding
TUG1	*APH1A*	protein_coding
TUG1	*PRRC2C*	protein_coding
TUG1	*NEK2*	protein_coding
TUG1	*YIPF4*	protein_coding
TUG1	*PROSER1*	protein_coding
TUG1	*NCOA5*	protein_coding
TUG1	*NCLN*	protein_coding
TUG1	*NUP214*	protein_coding

lncRNA	*Target*	Gene type
TUG1	*HP1BP3*	protein_coding
TUG1	*SPCS3*	protein_coding
TUG1	*DKC1*	protein_coding
TUG1	*DIAPH1*	protein_coding
TUG1	*TRAF7*	protein_coding
TUG1	*TPT1*	protein_coding
TUG1	*LDHA*	protein_coding
TUG1	*BTF3L4*	protein_coding
TUG1	*GDF11*	protein_coding
TUG1	*HNRNPA1*	protein_coding
TUG1	*RPS6*	protein_coding
TUG1	*HMGA1*	protein_coding
TUG1	*MTCH1*	protein_coding
TUG1	*BUD13*	protein_coding
TUG1	*SMAD6*	protein_coding
TUG1	*HNRNPA1L2*	protein_coding
TUG1	*ZFHX3*	protein_coding
TUG1	*MED9*	protein_coding
TUG1	*NPEPPS*	protein_coding
TUG1	*NARF*	protein_coding
TUG1	*WDR45B*	protein_coding
TUG1	*SH3GL1*	protein_coding
TUG1	*NECTIN4*	protein_coding
TUG1	*RPS27A*	protein_coding
TUG1	*MARCH6*	protein_coding
TUG1	*FADS1*	protein_coding
TUG1	*VPS26B*	protein_coding
TUG1	*PABPC3*	protein_coding
TUG1	*TIAL1*	protein_coding
TUG1	*UHMK1*	protein_coding
TUG1	*HNRNPU*	protein_coding
TUG1	*NPTN*	protein_coding
TUG1	*CUL4B*	protein_coding
TUG1	*PKNOX1*	protein_coding
TUG1	*RPL8*	protein_coding
TUG1	*SRSF2*	protein_coding
TUG1	*RNASEK-C17orf49*	protein_coding
TUG1	*TKT*	protein_coding
TUG1	*MELTF*	protein_coding
TUG1	*MAD2L1*	protein_coding
TUG1	*RHOBTB3*	protein_coding
TUG1	*AQP3*	protein_coding
TUG1	*SPRED1*	protein_coding
TUG1	*EEF2*	protein_coding
TUG1	*RPSA*	protein_coding
TUG1	*SPRY3*	protein_coding
TUG1	*CHRNA5*	protein_coding

lncRNA	*Target*	Gene type
TUG1	*HNRNPF*	protein_coding
TUG1	*RPS9*	protein_coding
TUG1	*BNC2*	protein_coding
TUG1	*TRMT112*	protein_coding
TUG1	*ATAD5*	protein_coding
TUG1	*EPS8L2*	protein_coding
TUG1	*MAF*	protein_coding
TUG1	*SKIDA1*	protein_coding
TUG1	*RAP2B*	protein_coding
TUG1	*EPHA10*	protein_coding
TUG1	*BTN3A2*	protein_coding
TUG1	*PTMA*	protein_coding
TUG1	*ZNF292*	protein_coding
TUG1	*ZNF470*	protein_coding
TUG1	*MCMBP*	protein_coding
TUG1	*MT-ND4*	protein_coding
TUG1	*RPL39*	protein_coding
TUG1	*RNU1-14P*	snRNA
TUG1	*AL021707.1*	pseudogene
TUG1	*CCNI2*	protein_coding
TUG1	*HACD2*	protein_coding
TUG1	*SNORA80E*	snoRNA
TUG1	*MT-RNR2*	Mt_rRNA
TUG1	*MT-RNR1*	Mt_rRNA
TUG1	*AC064799.1*	processed_pseudogene
TUG1	*TCTEX1D2*	protein_coding
TUG1	*KLHL23*	protein_coding
TUG1	*RPLP0P6*	processed_pseudogene
TUG1	*EMP2*	protein_coding
TUG1	*AC005005.1*	processed_pseudogene
TUG1	*PTMAP5*	transcribed_processed_pseudogene
TUG1	*AP000343.1*	processed_pseudogene
TUG1	*AL139100.1*	processed_pseudogene
TUG1	*RPL23P8*	processed_pseudogene
TUG1	*FTH1P8*	processed_pseudogene
TUG1	*AL161787.1*	processed_pseudogene
TUG1	*FTH1P10*	transcribed_processed_pseudogene
TUG1	*HNRNPA1P48*	protein_coding
TUG1	*FGD5-AS1*	antisense
TUG1	*TPI1P1*	processed_pseudogene
TUG1	*MTCO2P12*	unprocessed_pseudogene
TUG1	*TFAP2A-AS1*	antisense
TUG1	*RPS23P8*	processed_pseudogene
TUG1	*AC125238.2*	processed_pseudogene
TUG1	*SRRM1P3*	processed_pseudogene
TUG1	*MTCO1P12*	unprocessed_pseudogene
TUG1	*ACTG1P19*	processed_pseudogene

lncRNA	*Target*	Gene type
TUG1	*C1orf226*	protein_coding
TUG1	*RNA5-8S5*	rRNA
TUG1	*RBBP4P2*	processed_pseudogene
TUG1	*EEF1A1P4*	processed_pseudogene
TUG1	*RBBP4P1*	processed_pseudogene
TUG1	*RNY5*	misc_RNA
TUG1	*RALA*	protein_coding
TUG1	*MATR3*	protein_coding
TUG1	*RRAGD*	protein_coding
TUG1	*RSF1*	protein_coding
TUG1	*IPO5*	protein_coding
TUG1	*NTN1*	protein_coding
TUG1	*ZFAT*	protein_coding
TUG1	*TFRC*	protein_coding
TUG1	*ITM2A*	protein_coding
TUG1	*TNPO1*	protein_coding
TUG1	*FTL*	protein_coding
TUG1	*GRAMD1A*	protein_coding
TUG1	*HNRNPC*	protein_coding
TUG1	*BAMBI*	protein_coding
TUG1	*HSP90AB1*	protein_coding
TUG1	*ACOT7*	protein_coding
TUG1	*SCD*	protein_coding
TUG1	*EP300*	protein_coding
TUG1	*SNW1*	protein_coding
TUG1	*VAPA*	protein_coding
TUG1	*CENPT*	protein_coding
TUG1	*TSC2*	protein_coding
TUG1	*OAZ1*	protein_coding
TUG1	*TMEM147*	protein_coding
TUG1	*GRB10*	protein_coding
TUG1	*CCDC6*	protein_coding
TUG1	*YWHAE*	protein_coding
TUG1	*LPXN*	protein_coding
TUG1	*ATP5F1B*	protein_coding
TUG1	*FXR1*	protein_coding
TUG1	*SCAP*	protein_coding
TUG1	*KIAA2013*	protein_coding
TUG1	*MFN2*	protein_coding
TUG1	*BCAS2*	protein_coding
TUG1	*MED28*	protein_coding
TUG1	*CCNI*	protein_coding
TUG1	*CCND2*	protein_coding
TUG1	*IRF2BPL*	protein_coding
TUG1	*TCP1*	protein_coding
TUG1	*KHDRBS1*	protein_coding
TUG1	*TUBA1B*	protein_coding

lncRNA	*Target*	Gene type
TUG1	*STK35*	protein_coding
TUG1	*UQCR11*	protein_coding
TUG1	*PODXL*	protein_coding
TUG1	*PSMA1*	protein_coding
TUG1	*APOE*	protein_coding
TUG1	*PPFIA1*	protein_coding
TUG1	*H3F3B*	protein_coding
TUG1	*SPART*	protein_coding
TUG1	*ERG28*	protein_coding
TUG1	*EPC2*	protein_coding
TUG1	*SCRN1*	protein_coding
TUG1	*DBNL*	protein_coding
TUG1	*RSAD1*	protein_coding
TUG1	*PPM1B*	protein_coding
TUG1	*RPS24*	protein_coding
TUG1	*SINHCAF*	protein_coding
TUG1	*MFAP1*	protein_coding
TUG1	*IGFBP4*	protein_coding
TUG1	*ILF2*	protein_coding
TUG1	*RPS3*	protein_coding
TUG1	*ALDOA*	protein_coding
TUG1	*PLEKHG4B*	protein_coding
TUG1	*MARVELD1*	protein_coding
TUG1	*SLC16A1*	protein_coding
TUG1	*EEF1A1*	protein_coding
TUG1	*RNF166*	protein_coding
TUG1	*UBR1*	protein_coding
TUG1	*NAE1*	protein_coding
TUG1	*CBS*	protein_coding
TUG1	*LAPTM5*	protein_coding
TUG1	*ICA1L*	protein_coding
TUG1	*CTSB*	protein_coding
TUG1	*CCT2*	protein_coding
TUG1	*TMEM41B*	protein_coding
TUG1	*NDUFS5*	protein_coding
TUG1	*HNRNPA3*	protein_coding
TUG1	*ELP5*	protein_coding
TUG1	*TMEM43*	protein_coding
TUG1	*RPL38*	protein_coding
TUG1	*VANGL1*	protein_coding
TUG1	*MZT2A*	protein_coding
TUG1	*IMP3*	protein_coding
TUG1	*RPS12P23*	processed_pseudogene
TUG1	*FO393411.1*	processed_pseudogene
TUG1	*ZDHHC20*	protein_coding
TUG1	*SHMT2*	protein_coding
TUG1	*RPL27AP5*	processed_pseudogene

lncRNA	*Target*	Gene type
TUG1	*MTA1*	protein_coding
TUG1	*RIMBP3C*	protein_coding
TUG1	*SUMO3*	protein_coding
TUG1	*BRWD1*	protein_coding
TUG1	*EDARADD*	protein_coding
TUG1	*RPS23*	protein_coding
TUG1	*BLOC1S2*	protein_coding
TUG1	*EEF1A1P5*	processed_pseudogene
TUG1	*TUBB*	protein_coding
TUG1	*C5orf42*	protein_coding
TUG1	*CDC42SE1*	protein_coding
TUG1	*RPL37A*	protein_coding
TUG1	*ADH5*	protein_coding
TUG1	*ZNF649*	protein_coding
TUG1	*ZNF251*	protein_coding
TUG1	*PPIAP22*	processed_pseudogene
TUG1	*RNU1-134P*	snRNA
TUG1	*ZDHHC18*	protein_coding
TUG1	*BAG6*	protein_coding
TUG1	*RNVU1-7*	snRNA
TUG1	*SNORD15A*	snoRNA
TUG1	*RNU6-30P*	snRNA
TUG1	*RNVU1-1*	snRNA
TUG1	*HNRNPA1P40*	processed_pseudogene
TUG1	*RPS29*	protein_coding
TUG1	*EEF1A1P12*	processed_pseudogene
TUG1	*HNRNPA1P7*	processed_pseudogene
TUG1	*RN7SKP221*	misc_RNA
TUG1	*TMSB4XP4*	processed_pseudogene
TUG1	*HLA-DPB1*	protein_coding
TUG1	*RPS8P10*	unprocessed_pseudogene
TUG1	*HSP90AA2P*	processed_pseudogene
TUG1	*ATXN1L*	protein_coding
TUG1	*NDUFAF8*	protein_coding
TUG1	*MTND2P28*	unprocessed_pseudogene
TUG1	*AC067942.1*	transcribed_processed_pseudogene
TUG1	*RPL9P8*	pseudogene
TUG1	*RPL36AP39*	processed_pseudogene
TUG1	*RPL3P12*	processed_pseudogene
TUG1	*EEF1A1P6*	processed_pseudogene
TUG1	*TUBAP*	processed_pseudogene
TUG1	*RPL9P7*	processed_pseudogene
TUG1	*TMEM250*	protein_coding
TUG1	*AL133163.1*	processed_pseudogene
TUG1	*AC098831.1*	processed_pseudogene
TUG1	*RN7SL306P*	misc_RNA
TUG1	*SLC16A1P1*	processed_pseudogene

lncRNA	*Target*	Gene type
TUG1	*RPS23P1*	processed_pseudogene
TUG1	*ENO1P1*	transcribed_processed_pseudogene
TUG1	*RPL18P13*	processed_pseudogene
TUG1	*MTND5P11*	processed_pseudogene
TUG1	*AC024451.3*	processed_pseudogene
TUG1	*MTRNR2L1*	protein_coding
TUG1	*HMGB1P6*	processed_pseudogene
TUG1	*AC024619.4*	processed_pseudogene
TUG1	*RNA28S5*	rRNA
TUG1	*AC090004.1*	protein_coding
TUG1	*MTCYBP22*	processed_pseudogene
TUG1	*RNA18S5*	rRNA
TUG1	*CBSL*	protein_coding
TUG1	*BACE1-AS*	antisense
TUG1	*AC135068.6*	processed_pseudogene
TUG1	*C17orf49*	protein_coding
TUG1	*AL049873.2*	processed_pseudogene
TUG1	*AC011477.5*	processed_pseudogene
TUG1	*MTCO3P22*	processed_pseudogene
TUG1	*AC069257.3*	protein_coding
TUG1	*AC005258.1*	protein_coding
TUG1	*AD000090.1*	antisense
TUG1	*RNA18N5*	rRNA
