# Supplementary material for: The lncRNA MALAT1 is upregulated in urine of type 1 diabetes mellitus patients with diabetic kidney disease
Source: Genet Mol Biol. 2023 Jun 2;46(2):e20220291. doi: 10.1590/1678-4685-GMB-2022-0291 (PMC10240573; doi:10.1590/1678-4685-GMB-2022-0291)
Supplement: Table S2 - [file 1415-4757-GMB-46-2-e20220291-s3.pdf]

Genetics and Molecular Biology
DOI: https://doi.org/10.1590/1678-4685-GMB-2022-0291

Supplementary Material to "The lncRNA *MALAT1* is upregulated in urine of type 1 diabetes mellitus patients with diabetic kidney disease"

Table S2 - Significant KEGG pathways regulated by the target genes of the lncRNAs *MALAT1* and *TUG1*.

LncRNA *MALAT1*				
**Pathway Source**	**Pathway Name**	**p-value**	**q-value (FDR: BH-method)**	**q-value (Bonferroni)**
KEGG	Ribosome	0,00000000	0,00000000	0,00000000
KEGG	Parkinson disease	0,00000000	0,00000000	0,00000000
KEGG	Thermogenesis	0,00000000	0,00000000	0,00000000
KEGG	Oxidative phosphorylation	0,00000000	0,00000001	0,00000003
KEGG	Proteoglycans in cancer	0,00000000	0,00000010	0,00000049
KEGG	Pathogenic Escherichia coli infection	0,00000000	0,00000014	0,00000081
KEGG	Spliceosome	0,00000007	0,00000296	0,00002074
KEGG	Tight junction	0,00000009	0,00000323	0,00002583
KEGG	Leukocyte transendothelial migration	0,00000011	0,00000339	0,00003054
KEGG	MicroRNAs in cancer	0,00000059	0,00001545	0,00016991
KEGG	Cell cycle	0,00000055	0,00001585	0,00015851
KEGG	Adherens junction	0,00000092	0,00002211	0,00026528
KEGG	Herpes simplex infection	0,00000165	0,00003387	0,00047423
KEGG	Alzheimer disease	0,00000156	0,00003440	0,00044718
KEGG	Cellular senescence	0,00000194	0,00003480	0,00055680
KEGG	Epstein-Barr virus infection	0,00000187	0,00003569	0,00053536

LncRNA *MALAT1*				
**Pathway Source**	**Pathway Name**	**p-value**	**q-value (FDR: BH-method)**	**q-value (Bonferroni)**
KEGG	Phagosome	0,00000295	0,00004699	0,00084590
KEGG	Viral myocarditis	0,00000280	0,00004721	0,00080258
KEGG	Huntington disease	0,00000344	0,00005194	0,00098690
KEGG	Hippo signaling	0,00000362	0,00005196	0,00103917
KEGG	Cell adhesion molecules (CAMs)	0,00000496	0,00006782	0,00142426
KEGG	Focal adhesion	0,00000531	0,00006932	0,00152507
KEGG	Endocytosis	0,00000604	0,00007535	0,00173299
KEGG	Antigen processing and presentation	0,00001161	0,00013883	0,00333196
KEGG	RNA transport	0,00001261	0,00014471	0,00361766
KEGG	Fc gamma R-mediated phagocytosis	0,00001431	0,00015795	0,00410660
KEGG	Regulation of actin cytoskeleton	0,00001651	0,00016339	0,00473826
KEGG	Ribosome biogenesis in eukaryotes	0,00001549	0,00016460	0,00444428
KEGG	mRNA surveillance	0,00001629	0,00016701	0,00467621
KEGG	Vibrio cholerae infection	0,00002599	0,00024863	0,00745899
KEGG	Protein processing in endoplasmic reticulum	0,00003916	0,00036252	0,01123820
KEGG	RNA degradation	0,00007405	0,00066418	0,02125360
KEGG	Glycolysis / Gluconeogenesis	0,00008180	0,00071145	0,02347790
KEGG	Human cytomegalovirus infection	0,00012086	0,00102018	0,03468620
KEGG	Hepatocellular carcinoma	0,00014670	0,00120296	0,04210350
KEGG	Hepatitis C	0,00018026	0,00139826	0,05173580
KEGG	Bacterial invasion of epithelial cells	0,00017914	0,00142814	0,05141320
KEGG	Human T-cell leukemia virus 1 infection	0,00022472	0,00169720	0,06449380
KEGG	Oocyte meiosis	0,00041371	0,00304451	0,11873600
KEGG	PI3K-Akt signaling	0,00052020	0,00373240	0,14929600
KEGG	Arrhythmogenic right ventricular cardiomyopathy (ARVC)	0,00061079	0,00427554	0,17529700
KEGG	N-Glycan biosynthesis	0,00071525	0,00477390	0,20527800

LncRNA *MALAT1*				
**Pathway Source**	**Pathway Name**	**p-value**	**q-value (FDR: BH-method)**	**q-value (Bonferroni)**
KEGG	HIF-1 signaling	0,00070344	0,00480681	0,20188600
KEGG	Human immunodeficiency virus 1 infection	0,00090025	0,00587208	0,25837100
KEGG	Mitophagy - animal	0,00115973	0,00739650	0,33284300
KEGG	Shigellosis	0,00115973	0,00739650	0,33284300
KEGG	Viral carcinogenesis	0,00136769	0,00835164	0,39252700
KEGG	Non-homologous end-joining	0,00142075	0,00849490	0,40775500
KEGG	Kaposi sarcoma-associated herpesvirus infection	0,00147514	0,00864011	0,42336500
KEGG	Rap1 signaling	0,00180213	0,01014140	0,51721100
KEGG	Aminoacyl-tRNA biosynthesis	0,00179004	0,01027480	0,51374100
KEGG	Protein export	0,00196294	0,01083390	0,56336400
KEGG	Proteasome	0,00204723	0,01108590	0,58755500
KEGG	Salmonella infection	0,00243577	0,01294570	0,69906600
KEGG	Cysteine and methionine metabolism	0,00264645	0,01380970	0,75953100
KEGG	Non-alcoholic fatty liver disease (NAFLD)	0,00290829	0,01490500	0,83467900
KEGG	Allograft rejection	0,00342257	0,01693580	0,98227800
KEGG	AMPK signaling	0,00340655	0,01715230	0,97768000
KEGG	Fluid shear stress and atherosclerosis	0,00395622	0,01924470	1,00000000
KEGG	Cardiac muscle contraction	0,00416299	0,01991300	1,00000000
KEGG	Axon guidance	0,00475932	0,02203100	1,00000000
KEGG	Pathways in cancer	0,00471634	0,02219000	1,00000000
KEGG	Graft-versus-host disease	0,00577640	0,02631470	1,00000000
KEGG	Citrate cycle (TCA cycle)	0,00658476	0,02952850	1,00000000
KEGG	Pentose phosphate	0,00658476	0,02952850	1,00000000
KEGG	Type I diabetes mellitus	0,00731754	0,03182020	1,00000000
KEGG	Ubiquitin mediated proteolysis	0,00970423	0,04156890	1,00000000
KEGG	Platelet activation	0,01058440	0,04467240	1,00000000

LncRNA *TUG1*				
**Pathway Source**	**Pathway Name**	**p-value**	**q-value (FDR: BH-method)**	**q-value (Bonferroni)**
KEGG	Ribosome	0,0000000	0,0000000	0,0000000
KEGG	Spliceosome	0,0000000	0,0000004	0,0000008
KEGG	Biosynthesis of unsaturated fatty acids	0,0000516	0,0026305	0,0078915
KEGG	Alzheimer disease	0,0003571	0,0136604	0,0546417
KEGG	Pathogenic Escherichia coli infection	0,0008492	0,0259869	0,1299340
KEGG	HIF-1 signaling	0,0010547	0,0268941	0,1613650
KEGG	Thermogenesis	0,0019804	0,0432863	0,3030040
